# Phytochemicals from *Astragalus zederbaueri* as Acetylcholinesterase Inhibitors for Alzheimer’s Therapy

**DOI:** 10.1371/journal.pone.0346177

**Published:** 2026-04-10

**Authors:** Saman Asghar, Muhammad Umer Khan, Talha Jawaid, Raima Rehman, Abeeha Shafi, Abdullah R. Alzahrani, Zia Ur Rehman, Abida Khan

**Affiliations:** 1 Institute of Molecular Biology and Biotechnology, The University of Lahore, Lahore, Pakistan; 2 Department of Pharmacology, College of Medicine, Imam Mohammad Ibn Saud Islamic University (IMSIU), Riyadh, Saudi Arabia; 3 Centre of Excellence in Molecular Biology, University of the Punjab, Lahore, Pakistan; 4 Department of Pharmacology and Toxicology, Faculty of Medicine, Umm Al-Qura University, Makkah, Saudi Arabia; 5 Health Research Centre, Jazan University, Jazan, Saudi Arabia; 6 Department of Pharmaceutical Chemistry and Pharmacognosy, Faculty of Pharmacy, Jazan University, Jazan, Saudi Arabia; 7 Center For Health Research, Northern Border University, Arar, Saudi Arabia; Guru Nanak College, INDIA

## Abstract

Alzheimer’s Disease (AD), the dominant form of dementia that evolves with age, involves several mechanisms by which it progresses. The cholinergic hypothesis proposes that the activity of Acetylcholinesterase (AChE) causes the decline of cholinergic neurotransmission, which leads to Alzheimer’s Disease. Considerable studies are being conducted to determine the best AChE inhibitor. This study evaluated 40 phytocompounds from the *Astragalus zederbaueri* plant as potential AChE inhibitors for Alzheimer’s disease. The analysis of the phytochemicals was conducted using the control drug donepezil (co-crystallized ligand) through various computational tools and biological databases for docking, visualization, and simulation. The findings of our research showed that the key ligands according to the docking analysis were Rutin (AZ-29), Kaempferol-3-O-rutinoside (Nicotiflorin) (AZ-32), and Isoquercitrin (AZ-28); nevertheless, it was found that Rutin played the most effective role of an anti-AChE compound with an exceptional binding affinity of −15.043 kcal/mol. TRP341 and TYR286 were key amino acid residues in hydrogen bonds and π-π stacking interactions, respectively. The results of pharmacokinetic and toxicological analyses of these compounds were within the acceptable range. Moreover, the molecular dynamics simulation confirmed the stability of the complexes. Our findings suggest a novel phytochemicals from *Astragalus zederbaueri* for Alzheimer’s disease, paving the way for further experimental validation and drug development.

## Introduction

Alzheimer’s disease (AD) is generally the leading cause of dementia in the elderly community, characterized by the declining function of cholinergic neurons [1]. Epidemiological projections suggest that, if current trends continue, dementia prevalence in Europe may double over the next 30–35 years, while global cases could increase approximately threefold [2]. AD pathology begins years before clinical symptoms has supported updated frameworks for classifying AD neuropathological changes (ADNC) and staging disease progression from a preclinical phase to mild cognitive/behavioral impairment and ultimately AD dementia [3,4]. AD pathology is driven not only by cholinergic dysfunction but also by amyloid-β aggregation, tau hyperphosphorylation, synaptic loss, and neuroinflammation, all of which collectively contribute to progressive cognitive impairment [5,6].

In addition, nerve cells establish their interactions with other cells through collaborative mechanisms involving neurotransmitters. A critical aim of AD treatment is to target Acetylcholinesterase (AChE) because blocking AChE in cholinergic neurons plays a role in preventing synaptic depolarization and the destruction of AChE [7]. In the normal human brain, acetylcholinesterase (AChE) is responsible for about 80% of cholinesterase activity, while butyrylcholinesterase (BChE) is responsible for about 20%. However, as the neurodegenerative disease progresses, this distribution changes significantly, and BChE activity increases, while AChE activity decreases to approximately 10–15% of its normal level [8]. AChE inhibitors increase synaptic acetylcholine availability and can partially improve cognitive and behavioral symptoms by strengthening residual cholinergic signaling [9–11].

There are numerous natural cholinesterase inhibitors obtained specifically from plants. Due to insufficient potent and safe inhibitors, this diverse array of phytochemicals has assumed more importance in the ongoing efforts to find phytochemical-based medication for AD [12,13]. The flavonoid glycosides have proved to be such a potential candidate in the inhibition of acetylcholinesterase (AChE), which is a key enzyme responsible for the regulation of acetylcholine. It is assumed that these compounds bind to major residues in the AChE active site by hydrogen bonding and hydrophobic interaction, thus inhibiting the breakdown of acetylcholine. The glycoside group increases the solubility and bioavailability of flavonoids, which increases their cellular absorption and therapeutic outcomes. Also, they have antioxidant effects that provide a neuroprotective effect in reducing oxidative stress, one of the primary contributors to neurodegeneration. Together, these processes render flavonoid glycosides as a promising group of compounds to develop treatments against Alzheimer’s disease, in which AChE inhibitor and antioxidant properties are needed to manage symptoms and progression of the disease [6].

*Astragalus zederbaueri* is a medicinal plant known to produce a diverse array of secondary metabolites that are responsible for its pharmacological potential. The bioactive metabolites of *Astragalus zederbaueri* have a potential therapeutic implication on the treatment of cholinergic malfunction and oxidative stress changes that characterize the pathology of Alzheimer’s disease (AD). The capacity of plant-based phytochemicals in modulating the essential AD-related pathways, such as cholinesterase, antioxidant activities, and tau-related mechanisms, has been emphasized by previous studies, which validates the therapeutic potential of natural compounds in the treatment of AD [5]. Recent studies have reported that the ethyl acetate (EtOAc) extract of *Astragalus zederbaueri* has significant inhibitory activity against such enzymes as butyrylcholinesterase (BChE), which has an increasingly important role in the progression of Alzheimer’s disease (AD) as acetylcholinesterase (AChE) activity declines [14].

Previous approaches to drug discovery have been commercially successful in identifying new molecular entities, but getting from a lead compound to a drug that can be tested on patients takes over 12 years and around USD 1.8 billion. The term ‘*in silico*’ was recently appreciated for its efficiency in drug discovery regarding time, effort, and cost. Many new molecules have been identified using this method [15]. Several computational techniques have been developed to operate in parallel with experimental methods in improving compound screening [16]. Using computational biology, it has been observed that various classes of phytochemicals from plants have also been screened for significantly inhibitory activity against AChE. We, therefore, investigated the plant *Astragalus zederbaueri* and reported the phytochemicals that interact with the AChE in this research. Using docking and molecular simulation tools, we aimed to learn more about the importance of binding interactions of potentially novel molecules for the treatment of AD. Thus, phytochemicals may be proven effective for the inhibition of AD.

## Materials and methods

### Ligand retrieval and preparation

Forty phytochemicals reported from *Astragalus zederbaueri* were selected as ligands based on peer-reviewed phytochemical studies and supporting literature [14]. These compounds’ 3D structures were extracted from the PubChem database (https://pubchem.ncbi.nlm.nih.gov/) and saved in SDF Format.

Ligand preparation was performed using the LigPrep module in the Schrödinger Suite 2020−3 (Schrödinger, LLC, New York, NY, USA) [17]. All ligands were geometry-optimized using the OPLS3e force field. Ionization states were generated at pH 7.0 ± 2.0 using Epik, and relevant tautomers were retained. Stereoisomers were generated for compounds with undefined chiral centers, with the number of stereoisomers limited to a maximum of 32 per ligand to minimize redundancy. Default settings were applied for desalting, geometry minimization, and structure correction.

### Protein retrieval and validation

The targeted protein, i.e., AChE from *Homo sapiens* (PDB ID: 7E3H), was retrieved from the Protein Data Bank (PDB) [18], which is supported by the Research Collaboratory for Structural Bioinformatics (RCSB). The structure was selected according to its X-ray crystallographic data, and its resolution was 2.5Å. Structural validation was done with the help of PROCHECK [19], and it created a Ramachandran Plot. This revealed that 90.3% of the residues were in the favored regions, which is justification to the structural quality of the protein to conduct further computational studies.

### Protein preparation

The AChE structure was prepared with the help of the ‘Protein Preparation Wizard’ module in Maestro 12.5 [20]. This module identifies and eliminates any anomalies in its structure, incorporates hydrogen atoms into the structure, and optimizes the hydrogen bond network. Energy-based conformational sampling and rotamer optimization were used to fill side chains and complete loops using the PRIME function. Then, the compound structure was optimized with the help of PROPKA at pH 7.0 after preparation. During Protein preparation, all water molecules were eliminated because no conserved structural waters were involved in the interaction between the ligand and the AChE active site in the selected structure. Water removal provided an environment of simplified docking. The OPLS3e force field with default energy minimization criteria was used to minimize the structure.

### Grid generation

One of the significant aspects in molecular docking is the validation of the binding site. The centroid of the co-crystallized ligand within the AChE structure (PDB ID: 7E3H) was used to define the docking grid in Schrödinger Maestro [20]. The grid box was centered on the active site and coordinates were X: −43.37, Y: 37.73, and Z: −30.31 to fully encompass the native ligand. The interior box size was 10 x 10 x 10 Å and the exterior was 30 x 30 x 30 Å, ensuring that all of the binding pocket was covered.

### Molecular docking

The Glide module of Schrödinger Maestro was used to execute molecular docking [20]. Extra Precision (XP) mode was used in docking, wherein an advanced scoring feature was used to enhance precision. For each ligand, 10 poses were generated, and the pose with the lowest GlideScore was selected as the most favorable binding conformation. The grid cutoff of interaction per residue in the center of the grid was 12 Å. In order to test the reliability of docking, redocking of the co-crystallized ligand (donepezil) into the prepared protein structure was done, and the RMSD was obtained to confirm the accuracy of the docking protocol. The stability of the predicted ligand binding modes was determined by comparing key interactions within the docked complexes with the interactions observed in the co-crystallized structure.

### Visualization

Protein-ligand complexes visualization was also conducted to further gain insight into the interaction profiles and to justify the docking results. 2D interaction diagrams were generated using LigPlus version 2.2.8 (European Bioinformatics Institute, UK) [21]. The hydrogen bonds have been determined with a cutoff value of 3.5 Å of the donor acceptors and an angle cutoff of 30 degrees, which are standard values of the protein-ligand interaction. 3D analysis of binding poses, structural conformations and spatial orientation in the active site was analyzed using PyMOL version 3.0.5 (Schrodinger, LLC, New York, NY, USA) [22]. These visualization analyses gave supporting evidence on the docking findings through validation of the orientation, stability and viability of the ligands in the AChE ligand binding pocket.

### Structural interaction fingerprinting (SIFt) analysis

To determine the structural interactions of the selected ligands with the target protein, Structural Interaction Fingerprinting (SIFt) evaluation was performed with the help of Maestro [20]. The approach gives a comprehensive fingerprint of interactions including hydrogen bonding, hydrophobic interactions and other interaction-related molecular bindings that can be utilized in identifying the key binding functions. The obtained fingerprints were compared to draw conclusions regarding the similarities and differences between the ligand-protein interactions as well as make a choice in favor of the most appropriate candidates to continue the further examination.

### DFT studies (HOMO/LUMO analysis)

The Density Functional Theory (DFT) calculations were done using the Gaussian09 (Gaussian Inc., Wallingford, CT, USA) program, with GaussView version 5.0.8 serving as the graphical user interface [23]. The polarizable continuum model (PCM) was used to simulate the water solvent environment through complete optimization of the molecular geometries of the selected compounds in the B3LYP functional and using the basis set 6–311++G (d, p). Frontier molecular orbital (HOMO-LUMO) energies were determined to assess the reactivity and stability with a lower energy gap indicating higher reactivity. The molecular orbital distributions were analyzed to define the donor and acceptors regions of electrons. To further analyze the electronic nature, global reactivity descriptors; chemical hardness (η), softness (S), electronegativity (χ) and chemical potential (μ) were calculated based on the HOMO and LUMO values using the following equations to give more information on the nature of the compounds and their therapeutic capacity.:


$$\eta \;=\frac{\text{E}(\text{LUMO})\;-\text{ E}(\text{HOMO})}{\text{2}}$$

$$\text{S}=\frac{\text{1}}{\text{2}\eta }$$

$$\chi =-\frac{\text{E}(\text{HOMO})\;+\text{ E}(\text{LUMO})}{\text{2}}\;$$

μ=−χ

### Pharmacokinetic and toxicological analyses

Pharmacokinetic properties of the selected compounds were predicted using the SwissADME web server [24] to assess absorption, distribution, metabolism, and excretion (ADME) profiles. In addition, the pkCSM web server [25] was used to estimate pharmacokinetic attributes including intestinal absorption, volume of distribution (VDss), Cytochrome P450 enzyme inhibition, total body clearance, and AMES mutagenicity. Acceptable ranges were defined based on established literature cut-offs, such as high intestinal absorption (>30%), favorable log VDss (>0.45), and non-inhibitory behavior toward major CYP isoforms (CYP3A4, CYP2D6).

For toxicity evaluation, two complementary web-based tools were used: ProTox-II [26] and StopTox [27]. ProTox-II provided predictions for acute toxicity (LD50), hepatotoxicity, cytotoxicity, carcinogenicity, mutagenicity, immunotoxicity, and toxicity targets, integrating fragment propensities, molecular similarity, and machine-learning models. StopTox, a QSAR-based platform, was applied to further assess toxicity endpoints, relying on curated and integrated models from the largest open-source toxicity databases. Together, these tools provided a robust prediction of pharmacokinetic and toxicological safety profiles of the tested compounds.

### Molecular dynamics (MD) simulations

To determine conformational stability of AChE in complexes with the ligands of interest, MD simulations were conducted. The AMBER force field was used to prepare the systems, and each protein-ligand complex was placed in a TIP3P water box with periodic boundary conditions. Neutralization was done with counter ions. A slow heating to 300 K was then done followed by the minimization of energy, and equilibration was performed under both NVT and NPT ensembles. The production simulation was run for 100 ns at a time step of 2 fs, and the trajectories were periodically saved every 10 ps. Long-range electrostatics were solved using the Particle Mesh Ewald (PME) method, but bond lengths of hydrogen atoms were kept constant using the SHAKE algorithm. Temperature and pressure were controlled using the Langevin thermostat and Berendsen barostat, respectively. Trajectory analyses, including RMSD, RMSF, radius of gyration (Rg), and hydrogen bond occupancy, were carried out using AMBER tools [28].

## Results

### Protein retrieval and structure validation

The crystal structure of AChE (*Homo sapien)* was taken from Protein Data Bank with the accession number 7E3H. Due to the high resolution of the structure, it was selected for molecular docking.

**Fig 1:**
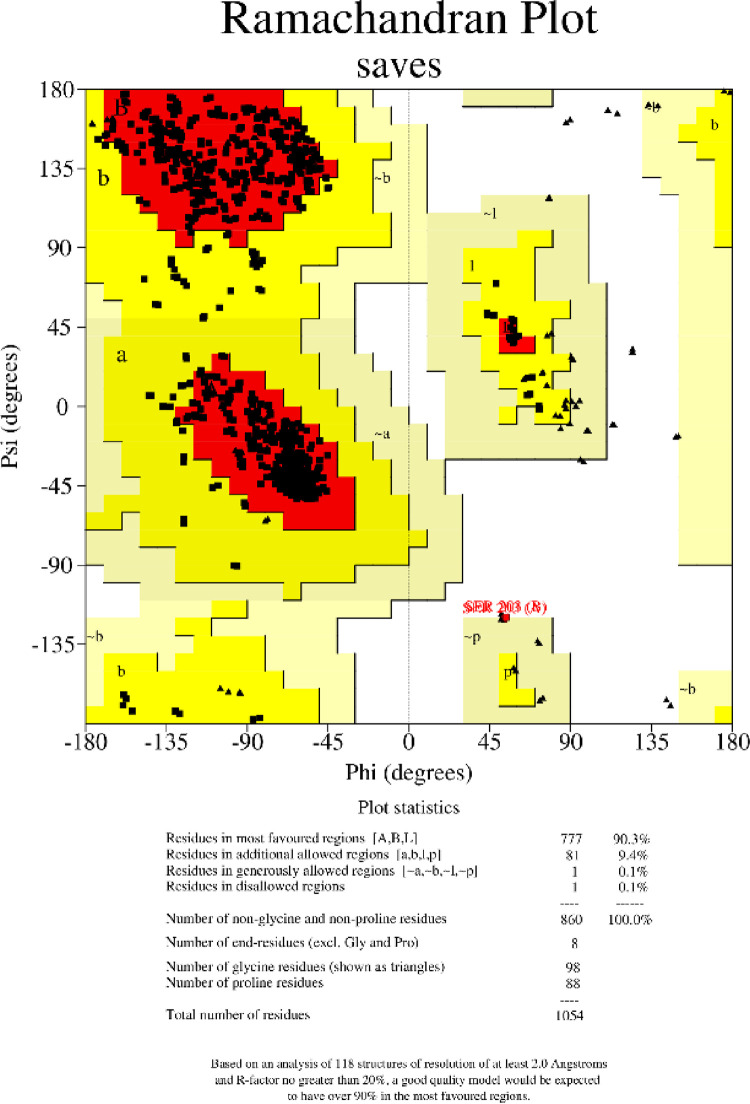
Ramachandran Plot showing 90.3% of residues in favored regions, 9.4% in allowed regions, and 0.1% in disallowed regions. The plot indicates good overall protein structure quality.

The Ramachandran plot studied the protein’s stereochemical quality through evaluation of the distributions of phi (Φ) and psi (Ψ) angles across its amino acid residues. The findings shown in Fig 1 suggest that 90.3% of the residues (777 residues) reside in the most favored regions, indicating that the general protein structure is nicely folded and of superior quality. In addition, 9.4% of the residues (81 residues) fall into the allowed region, which is standard and acceptable for protein models, although some residues deviate a little from ideal conformations. There is only 1 residue (0.1%) within the broadly permitted regions, and 1 residue (of the same 0.1%) exists in the disallowed region. The residue in the disallowed area is Serine 203 (A). The structure of the protein also comprises 98 glycine residues, which are flexible and likely to emerge in regions less commonly found on the plot, and 88 proline residues, which are constrained by their cyclic nature to certain conformational areas. Ultimately, the plot shows that the protein structure meets anticipated stereochemical requirements, with moderate deviations, suggesting a quality model with spots for possible modification.

### Ligands retrieval

In this study, 40 bioactive phytocompounds of *Astragalus zederbaueri* were taken through a literature review from the database for molecular docking studies for AChE inhibition. The complete information on these compounds, including 2D and 3D structures as well as SMILES notation, is provided in Supplementary S1 Table. The PubChem CIDs and Date of retrieval of all compounds are mentioned in Supplementary S2 Table.

### Molecular docking

The results show that the co-crystal ligand (CCL) has the highest binding affinity for the target protein, with the lowest Glide Score of −17.062.(Supplementary S1 Fig) The top three ligands following CCL—AZ-29, AZ-32, and AZ-28 exhibit docking scores of −15.043, −13.935, and −12.437, respectively. In contrast, AZ-24 has the highest docking score of −0.812, indicating weak binding affinity to AChE.

### Docking validation

**Fig 2:**
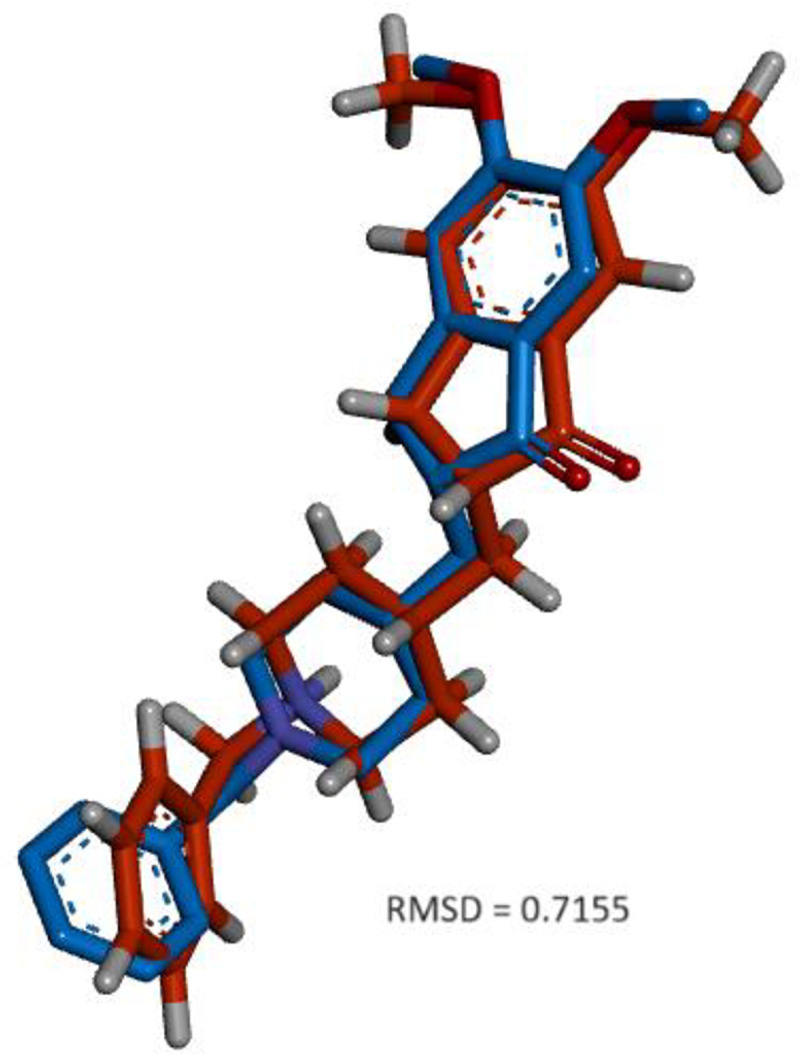
Structural alignment of two molecular conformers, depicted in blue and red, with an RMSD (Root Mean Square Deviation) value of 0.7155, indicating the degree of similarity between their spatial configurations.

Fig 2 compares the binding poses of the ligand donepezil within AChE. It overlays the experimentally determined structure of donepezil bound to AChE (red, PDB ID 7E3H) with the conformation obtained through molecular docking (blue). The RMSD (Root Mean Square Deviation) value of 0.7155 indicates close alignment between the docked ligand and the experimental structure, suggesting that the docking simulation accurately predicted donepezil’s binding pose within AChE (RMSD < 2.0 Å is generally considered acceptable for validation).

### Visualization of docked complexes

**Fig 3:**
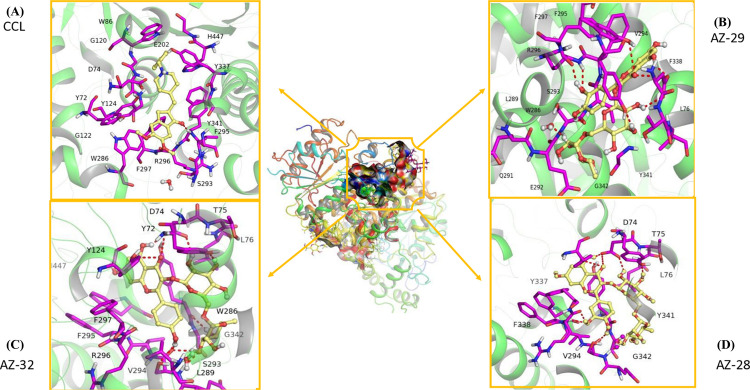
3D visualization of ligands (A) CCL, (B) AZ-29, (C) AZ-32, and (D) AZ-28 with AChE drawn by using PyMOL. These structures show the binding interaction between the ligands and the residues of amino acids within the binding pocket of the AChE protein.

**Fig 4:**
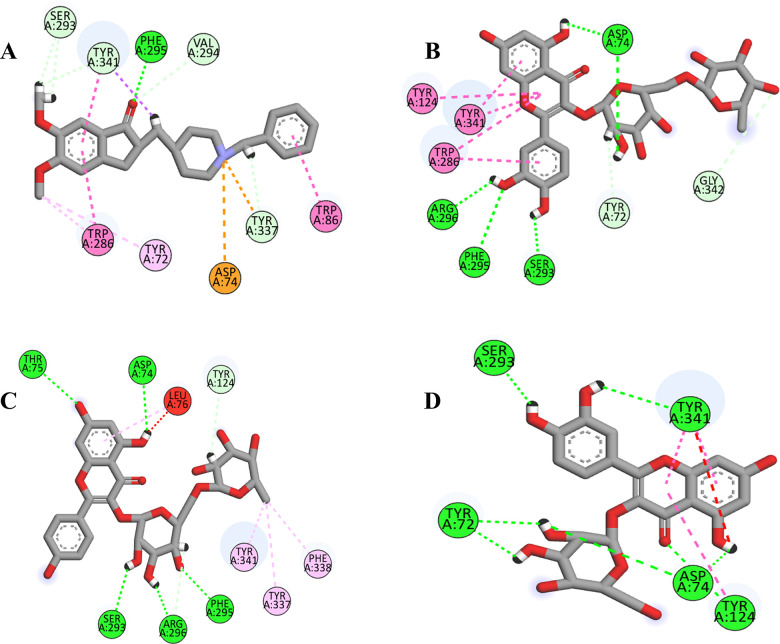
2D interaction diagrams of four ligands (A) CCL, (B) AZ-29, (C) AZ-32, and (D) AZ-28 bound to the target protein structure 7E3H. Each ligand’s interactions with surrounding amino acid residues are shown, with red arcs indicating hydrophobic interactions and green dashed lines representing hydrogen bonds.

Visualization of molecular docking results for the chosen four ligands depicted in Figs 3 and 4, included CCL, AZ-29, AZ-32, and, AZ-28, based on the top GlideScores that arise when the ligands are docked at the acetylcholinesterase (AChE) active site, in terms of binding affinity and stability. CCL has a high binding affinity with PHE291, SER293 and TYR341 as well as strong π-π stacking interactions with TYR286 and TRP86 (Fig 3A). AZ-29 exhibits the most extensive interactions, showing strong binding through hydrogen bonds with ASP74, SER293, PHE295, ARG296 and GLY342, as well as π-π stacking with TRP286, TYR124 and TYR341 (Fig 3B). AZ-32 also demonstrates high interaction with ASP74, SER293, PHE295, ARG296 by forming hydrogen bonds but lack π-π stacking, make it slightly less interactive than AZ-29 (Fig 3C). AZ-28 has fewer interactions but maintains moderate binding stability through hydrogen bonds (Fig 3D). Among the compounds, AZ-29 shows the best score when bound to AChE, while AZ-32, and AZ-28 also demonstrate good interactions but are less potent.

Fig 4 (A-D) presents the 2D interaction profiles of the same four ligands with AChE. AZ-29 again demonstrates strong binding energy through multiple hydrogen bonds and π–π stacking interactions, resulting in a slightly better stability than CCL. Although AZ-32 makes several favorable contacts, it shows a higher (less favorable) docking score than AZ-29. AZ-28, with fewer interactions overall, exhibits moderate affinity and yields the least favorable docking score among the ligands. These results consistently highlight AZ-29 as the most potent binder, followed by AZ-32, while AZ-28 shows weaker binding.

**Fig 5:**
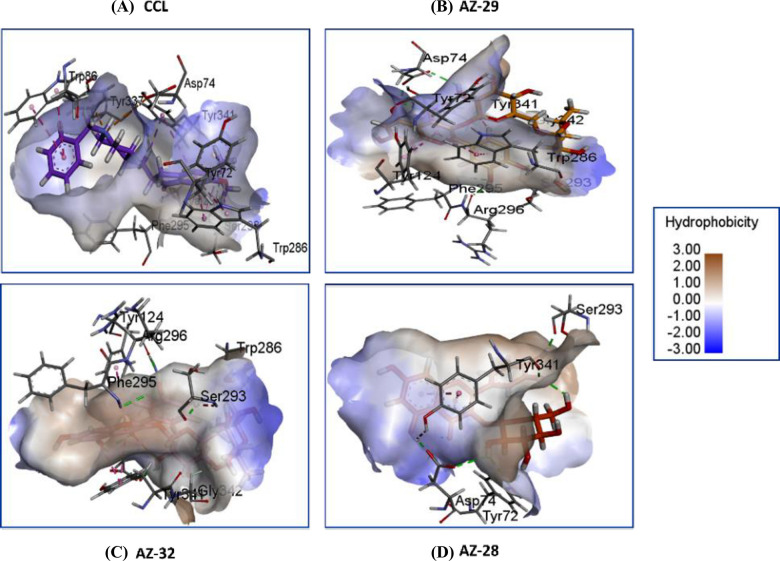
Hydrophobicity surface maps of (A) CCL, (B) AZ-29, (C) AZ-32, and (D) AZ-28 showing key interactions with residues such as Tyr72, Asp74, Phe295, Trp286, and Tyr341 within the binding pocket of AchE.

A color-coded hydrophobicity map of the ligands attached in the active site of AChE is shown in Fig 5. The scale has been used to indicate brown and blue as hydrophobic and hydrophilic regions, respectively. CCL binds to TYR72, ASP74, TRP286, and TYR86, and forms hydrogen bond in the hydrophilic loops (PHE295, TYR341) and strong hydrophobic interactions in nonpolar loops (PHE295, TRP286). AZ-29 is bound to TYR341, TYR124 and TRP286, in hydrophobic region and residues in hydrophilic region involved in polar interactions are, ASP74, PHE295, ARG296 and SER293. AZ-32 interacts with TYR124, ARG296, PHE295, SER293, and TRP286 and has polar contacts with SER293, ARG296, PHE295, ASP74 and has interactions in hydrophobic region with residues PHE338 and TYR341. AZ-28 reacts with TYR341, ASP74, TYR72, and SER293, entailing hydrogen bonds. Together, these ligands bind to a shared binding pocket mostly with hydrophobic and hydrophilic contacts, and PHE295, TRP286, and ASP74 form the major residues whose presence is essential to the binding of the ligand to the protein.

### Structural interaction fingerprinting (SIFt) analysis

**Fig 6:**
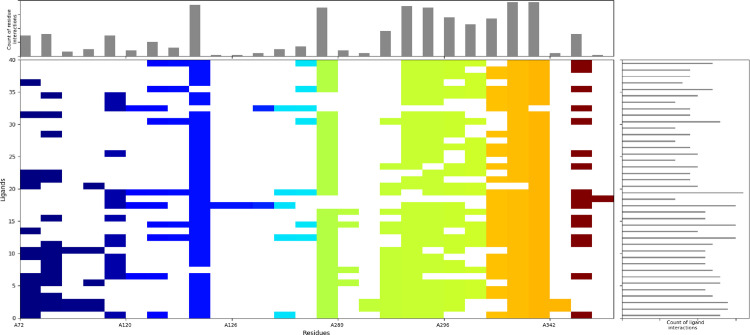
Fingerprint analysis of the docked complexes of AChE with the phytochemicals illustrating the count of residue interactions and the count of ligand interactions.

SIFt analysis of AChE is displayed in Fig 6 with all the docked complexes of phytochemicals. All ligands were docked in the active site of the AChE protein. The count of residue interaction has shown that TYR 124, TRP 286, VAL 294, PHE 295, PHE 338, and TYR 341 residues were able to interact with almost 80% of ligands docked into the active sites. This indicates that these 6 residues are hotspots in the binding cavity of the AChE protein and can help in accommodating potential inhibitors of the AChE protein.

The count of ligand interactions has shown that AZ-29 and AZ-32 have over 70% of the interactions. Both of these ligands have a Glide GScore of −15.04 and −13.935, showing that they have the potential for inhibition.

**Fig 7:**
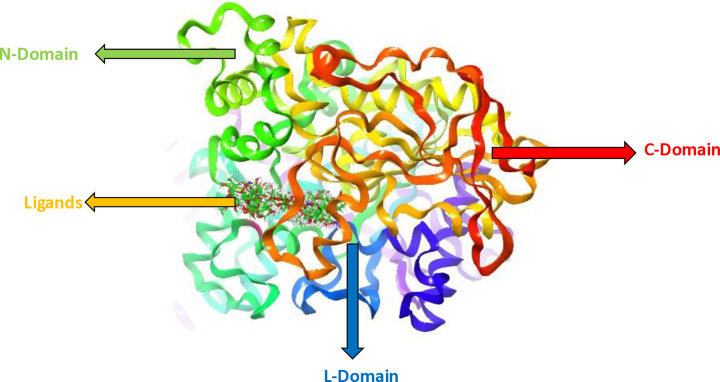
Protein structure of AChE with all the docked ligands along with different domains of the protein structure.

Fig 7 represents the structure of the AChE protein with all the docked ligands. Stabilizing the overall conformation as well as allowing molecular interactions, the N-terminal domain (green region) of the structure plays a main role. Key structural elements are linked by the linker (L) domain (yellow, green, and blue regions), showing that the catalytic residues are correctly positioned and the interactions within the active site are stabilized. Of the enzyme, the C-terminal domain (orange and red regions in the image) is also important for maintaining structural integrity, essential for enzyme function overall. At the center is the ball and stick structure depicting 40 ligands of the binding pocket.

**Fig 8:**
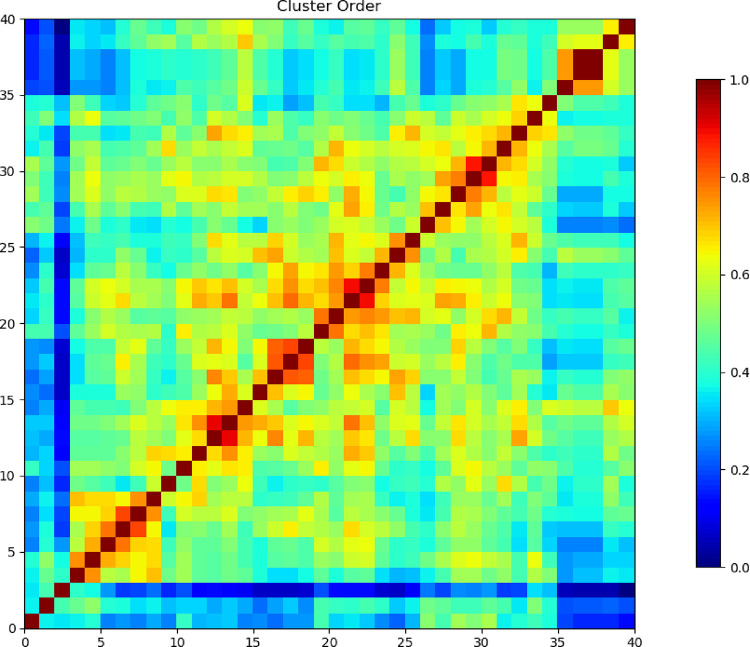
Heat map showing Cluster Order of docked complexes of AChE protein with 40 phytochemicals. The red diagonal line indicates high similarity, while the blue lines indicate low similarity.

The heatmap shown in Fig 8 illustrates the clustering of AChE with 40 phytochemicals extracted from a plant. The diagonal red line indicates that each docked complex shares high self-similarity, as expected, representing a consistent docking conformation within the clusters. The smaller off-diagonal red and yellow squares signify clusters of complexes with similar binding conformations, indicating that certain phytochemicals adopt comparable interaction profiles within the active site of AChE. On the contrary, the blue areas indicate low similarity, implying that a large variety of binding modes of certain phytochemicals can occur, indicating that these compounds can be bound to AChE in different conformations. The color gradient, ranging from blue (low similarity) to red (high similarity), quantifies the degree of clustering. It is to be noted that although there are phytochemicals that have similar binding modes with AChE, others have various conformational flexibilities, which may indicate multiple binding sites or poses on the enzyme.

### DFT studies (HOMO/LUMO analysis)

The density functional theory (DFT) analysis of four compounds reveals significant insights into their electronic properties and potential as drug candidates. The compounds CCL, AZ-29, AZ-32, and AZ-28 show better electronic characteristics and thus can be used for therapeutic purposes.

**Fig 9:**
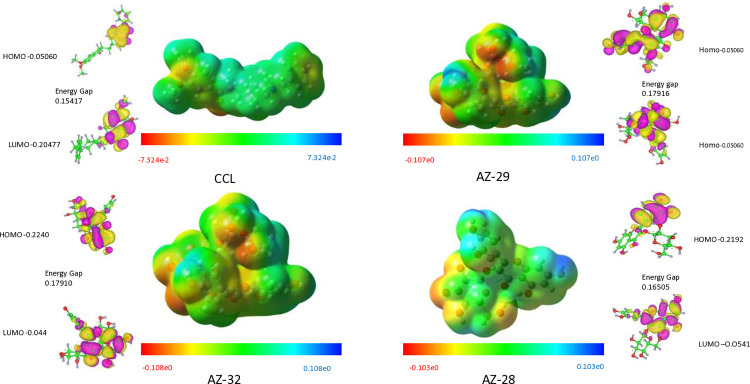
Highest Occupied Molecular Orbital and Lowest Unoccupied Molecular Orbital (HOMO/LUMO) of CCL, AZ-29, AZ-32, and AZ-28, with corresponding energy gaps. The color scale represents the electrostatic potential distribution, and the HOMO-LUMO orbitals are shown with energy values indicating electron distribution and reactivity.

Table 1 shows the 12.4713 dipole moment for AZ-32, it can be extrapolated that compounds with higher dipole moments enable stronger solvent-solute interactions in polar solvents and then can be regarded as having better bioavailability. The HOMO and LUMO values suggest that the best compounds are those with electronic configurations suitable for drug intercalation; the calculated energy difference (ΔE) should be small for increased reactivity. More explicitly, as can be seen, both in Table 1 and Fig 9, the energy gap of CCL and AZ-29 is smaller (0. 15417 eV), and it proposed that the studied compound could be more effective in biological systems.

**Table 1:** DFT Analysis of Ligands: Electronic Properties Including Dipole Moment, HOMO-LUMO Gap, Ionization Potential, and Reactivity Descriptors.

Parameters for DFT analysis											
Ligand	Dipole moment (Debye)	HOMO (a.u.)	LUMO (a.u.)	Energy Gap (ΔE_Gap_)	Ionization Potential (eV)	Electron affinity (eV)	Electronegativity χ (eV)	Electrochemical potential μ (eV)	Hardness η (eV)	Softness S (eV)-1	Electrophilicity ω (eV)
CCL	4.9515	−0.20477	−0.05060	0.15417	0.20477	0.05060	0.12768	−0.12768	0.07709	12.95	0.105
AZ-29	11.0036	−0.22380	−0.04464	0.17916	0.22380	0.04464	0.13422	−0.13422	0.08958	11.16	0.100
AZ-32	12.4713	−0.22400	−0.0449	0.17910	0.22400	0.04490	0.13495	−0.13495	0.08955	11.16	0.101
AZ-28	7.2143	−0.21920	0.05415	0.16505	0.21920	0.05415	0.13618	−0.13618	0.082525	12.12	0.112

Additionally, the values of ionization potentials and electron affinity are higher in the more promising compounds, which suggests a better possibility of electron transfer which is important for the agent’s activity. Other values of electronegativity and electrochemical potential also define the stability as well as the reactivity of these compounds.

Therefore, the results of the study show that CCL, AZ-29, AZ-32, and AZ-28 are characterized by suitable electronic properties that may contribute to further work on the drug creation. This points the attention towards the second part of the electronic properties which shows how effective a particular drug candidate is most likely to be.

The ESP maps demonstrate the distribution of the surface charges with red areas depicting the negative potential and blue areas depicting the positive potential, respectively. This indicates that the nucleophilic and electrophilic attacks may occur in different places. These findings demonstrate differences in reactivity between the compounds as indicated by the difference in HOMO-LUMO gaps and charge distributions (Fig 9). In general, HOMO-LUMO analysis and ESP mapping are useful in understanding the electronic structures, stability, and possible chemical behavior of the molecules being studied.

### Pharmacokinetics

Pharmacokinetic profiles of selected phytochemicals (CCL, AZ-29, AZ-32, AZ-28), each with the potential for being an AD treatment candidate are presented in Supplementary S3 Table. All compounds have low water solubility, requiring formulation change to improve bioavailability, with CCL showing the least solubility. However, as indicated by its moderate permeability through intestinal cells, AZ-28 possesses effective absorption potential. The compounds are P-glycoprotein substrates, and CCL is the only compound that inhibits this transporter potently, thereby enhancing therapeutic concentrations of the compound in the brain. AZ-29 appears to be distributed broadly with the highest volume compared with AZ-28 and AZ-32 with lower volumes which may target specific tissues. Notably AZ-32 and AZ-29 have higher unbound fractions in circulation, suggesting higher pharmacological activity at the site of action than CCL. As regards blood-brain barrier (BBB) permeability CCL has a moderate potential to permeate the brain, and AZ-29 and AZ-28 have BBB permeability values that are tolerable and can be improved by the use of delivery strategies. A difference in its metabolic profiling, CCL is susceptible to interactions with CYP2D6 and CYP3A4 enzymes, whereas the other compounds exhibit simpler metabolic profiles. The excretion rates of AZ-28 and CCL from the body are rapid, minimizing toxicity risks, and AZ-29 and AZ-32 have slower elimination rates, so the effects are long-lasting. All compounds are also safe in terms of toxicity, except CCL, whose hepatotoxicity precludes its use in long-term AD patients in favor of non-hepatotoxic ones such as AZ-29.

The analysis of organ toxicity and toxicity endpoint of the ligands CCL, AZ-29, AZ-32, and AZ-28 indicated a number of results (Table 2). All ligands exhibited an inhibitory potential in hepatotoxicity, with CCL being the lowest at 0.98, AZ-28 at 0.82, and AZ-29 and AZ-32 with 0.80 each, and it is indicating minimal risk. On carcinogenicity, AZ-29, AZ-32, and AZ-28 scored 0.91, 0.91, and 0.85, respectively, implying that they were less likely to cause cancer than CCL, which scored 0.50. All the ligands were high in immunotoxicity, which reflects a strong immune support. The mutagenicity analysis indicated that AZ-29, AZ-32, and AZ-28 had the scores of 0.88, 0.88, and 0.76, respectively, and these scores represented lower mutagenic risk in comparison with the score of CCL, which was 0.53. The cytotoxicity was found to be acceptable with a range of 0.64 to 0.69 in all the ligands. Finally, in Tox21 Nuclear Receptor Signaling Pathways, the ligands had enormous inhibitory effects whose scores were greater than 0.90. These results highlight the good safety profiles of AZ-29 and AZ-32 as promising agents for future therapeutic studies (Table 2).

**Table 2:** Pro-Tox II toxicological parameters of the phytoconstituents of *Astragalus zederbaueri* that showed the high inhibitory effects.

Classification	Target	CCL		AZ-29		AZ-32		AZ-28	
		Pre	Pro	Pre	Pro	Pre	Pro	Pre	Pro
**Organ toxicity**	**Hepatotoxicity**	I	0.98	I	0.80	I	0.80	I	0.82
**Toxicity endpoints**	**Carcinogenicity**	A	0.50	I	0.91	I	0.91	I	0.85
	**Immunotoxicity**	A	0.95	A	0.98	A	0.95	A	0.66
	**Mutagenicity**	I	0.53	I	0.88	I	0.88	I	0.76
	**Cytotoxicity**	A	0.63	I	0.64	I	0.64	I	0.69
**Tox21-Nuclear receptor signaling pathways**	**AhR**	I	0.81	I	0.83	I	0.83	I	0.92
	**AR**	I	0.96	I	0.98	I	0.98	I	0.90
	**AR-LBD**	I	0.99	I	0.99	I	0.99	I	0.98
	**Aromatase**	I	0.89	I	0.99	I	0.99	I	1.0
	**ER**	A	0.58	I	0.95	I	0.95	I	0.91
	**ER-LBD**	I	0.98	I	0.99	I	0.99	I	0.99
	**PPAR-Gamma**	I	0.99	I	0.98	I	0.98	I	0.99
	**nrf2/ARE**	I	0.98	I	0.99	I	0.99	I	0.98
**Tox21-Stress response pathways**	**HSE**	I	0.98	I	0.99	I	0.99	I	0.98
	**MMP**	I	0.88	I	0.97	I	0.97	I	0.98
	**p53**	I	0.99	I	0.90	I	0.90	A	0.50
	**ATAD5**	I	0.99	I	0.99	I	0.99	I	1.0

Pre: prediction; Pro: probability; A: active; I: inactive; AhR: Aryl hydrocarbon Receptor; AR-LBD: Androgen Receptor Ligand Binding Domain; ER-LBD: Estrogen Receptor Ligand Binding Domain; PPAR-Gamma: Peroxisome Proliferator Activated Receptor Gamma (PPAR-Gamma); nrf2/ARE: Nuclear factor (erythroid-derived 2)-like 2/antioxidant responsive element (nrf2/ARE); HSE: Heat shock factor response element; MMP: Mitochondrial Membrane Potential; p53: Phosphoprotein (Tumor Suppressor); ATAD5: ATPase family AAA domain-containing protein 5.

CCL has the lowest molecular weight (380), while AZ-29 has the highest (610), reflecting a size diversity that may be advantageous for targeting different biological systems (Supplementary S2 Fig). All compounds display a low number of rotatable bonds (4–6), suggesting good conformational rigidity, which is often associated with enhanced binding affinity. Most compounds show minimal violations of Lipinski’s Rule of Five, with AZ-28 having only two, indicating overall favorable oral bioavailability. Balanced hydrogen bond donor and acceptor counts further support their drug-like characteristics, while logP values suggest moderate hydrophobicity consistent with good membrane permeability. Synthetic accessibility scores range from 3.4 to 6.5, with CCL (3.4) being the easiest to synthesize. Bioavailability scores of 0.17 for AZ-32 and AZ-28, and 0.55 for CCL, indicate promising oral bioavailability, reinforcing their potential as candidates for further drug development.

Analysis of the toxicity and irritation potential of different ligands was generated and acquired through various routes of exposure. As shown in Table 3, acute inhalation and dermal toxicity studies show CCL falls under the non-toxic category, while in oral toxicity, the compound was identified as toxic. Further, it was non-irritating to the eyes, and it was devoid of skin sensitization effect, skin irritation, and skin corrosion. However, AZ-29 was non-toxic upon inhaled and oral administration but classified as toxic upon dermal toxicity prediction. As with CCL, AZ-29 had no impact on eye irritation and was also rated as a non-sensitizer indicating negative for skin irritation and corrosion. Toxicological analysis of acute inhalation and oral toxicity of AZ-32 and AZ-28 was non-toxic, but toxic results were obtained when they were checked for dermal toxicity. Both ligands failed to cause eye irritation and were non-sensitizers using skin irritation and corrosion results. With equal concentrations of all ligands being non-irritant through inhalation, the dermal toxicity was not constant which calls for route-based evaluations of toxicity.

**Table 3:** StopTox toxicity parameters of the selected phytochemicals of *Astragalus zederbaueri.*

Ligands	Acute Inhalation Toxicity	Acute Oral Toxicity	Acute Dermal Toxicity	Eye Irritation and Corrosion	Skin Sensitization	Skin Irritation and Corrosion
CCL	Non-Toxic	Toxic	Non-Toxic	Non-Toxic	Non-Toxic	Negative
AZ-29	Non-Toxic	Non-Toxic	Toxic	Non-Toxic	Non-Sensitizer	Negative
AZ-32	Non-Toxic	Non-Toxic	Toxic	Non-Toxic	Non-Sensitizer	Negative
AZ-28	Non-Toxic	Non-Toxic	Toxic	Non-Toxic	Non-Sensitizer	Negative

The bioactivity profiles of CCL, AZ-29, AZ-32, and AZ-28 are displayed in Table 4. CCL achieved a GPCR ligand score of 0.22 and significant enzyme inhibition at 0.25 but had negative scores for ion channel modulation (−0.14) and kinase inhibition (−0.16). AZ-29 recorded a GPCR score of −0.05 with moderate enzyme inhibition at 0.12, despite a low ion channel modulation score of −0.52. AZ-32 had a GPCR score of −0.01 and a negative ion channel modulation score of −0.43 while achieving an enzyme inhibition score of 0.18. In contrast, AZ-28 showed the most favorable profile with a GPCR score of 0.06, nuclear receptor ligand activity at 0.20, and strong enzyme inhibition at 0.42.

**Table 4:** Bioactivity score values of the selected phytochemicals assessed by the online tool Molinspiration.

Phytocompounds	Parameters of Bioactivity Score					
	GPCR ligand	Ion channel Modulator	Kinase Inhibitor	Nuclear Receptor ligand	Protease Inhibitor	Enzyme Inhibitor
**CCL**	0.22	−0.14	−0.16	0.03	0.03	0.25
**AZ-29**	−0.05	−0.52	−0.14	−0.23	−0.07	0.12
**AZ-32**	−0.01	−0.43	−0.09	−0.17	−0.04	0.18
**AZ-28**	0.06	−0.04	0.13	0.20	−0.06	0.42

While AZ-29 and AZ-32 exhibited weaker GPCR and ion channel activities, their moderate enzyme inhibition suggests therapeutic potential, especially in enhancing cholinergic function for AD treatment. AZ-28 stands out with superior activity across multiple parameters, indicating its strong potential as an AChE inhibitor.

AZ-29 was selected for molecular dynamics (MD) simulations as it exhibited the most favorable docking score among all screened compounds. Additionally, its acceptable ADMET profile and DFT analysis further highlighted its structural stability and favorable reactivity toward acetylcholinesterase (AChE), justifying its selection for MD studies.

### MD simulation

#### RMSD.

**Fig 10:**
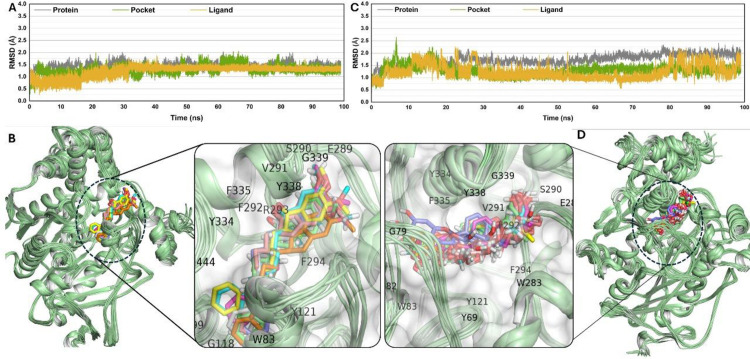
RMSD values of CCL and AZ-29 (A and C) and also the Residue interaction of Ligands with AChE protein on different possible poses (B and D).

In Fig 10, A and C display Root Mean Square Deviation (RMSD) plots of CCL and AZ-29 respectively, over time for three different entities: the protein backbone, the binding pocket, and the ligand. The gray lines indicate the RMSD of the entire protein backbone, and the fact that the RMSD remains at a relatively stable level, oscillating around a specific point, suggests that the overall protein structure of the system remains stable during the simulation. The green lines represent the RMSD of the residues within the binding pocket. The orange lines show the RMSD of ligands; lower and more stable values of ligand RMSD imply that the ligand remains in a consistent orientation within the pocket.

### Protein-ligands interaction

In Figs 10 B and D, the structural poses retrieved from the molecular dynamics (MD) simulations are shown highlighting the ligand-protein interactions of CCL and AZ-29 respectively. In Fig 10 B, an enlarged view of the binding site is shown, where the protein is represented in cartoon form and colored green, while multiple conformations or poses of the ligand in different colors are also superimposed. Likewise, Fig 10 D displays another set of ligand conformations in the molecular dynamics simulation as shown below with the binding pocket circled by a dashed line.

The overlay of the ligands in both figures implies that these ligands bind in the same orientation throughout the simulation and are firmly bound. These include key interactions with residues, namely TYR 338, PHE 335, and VAL 291 for stability. Besides, the ligand interactions with other residues such as ARG293, TYR334, and PHE294 are illustrated; thus, hydrogen bonds, hydrophobic interactions, or π-π stacking between the residues and the ligands seem to play central roles in the binding and selectivity.

#### RMSF.

**Fig 11:**
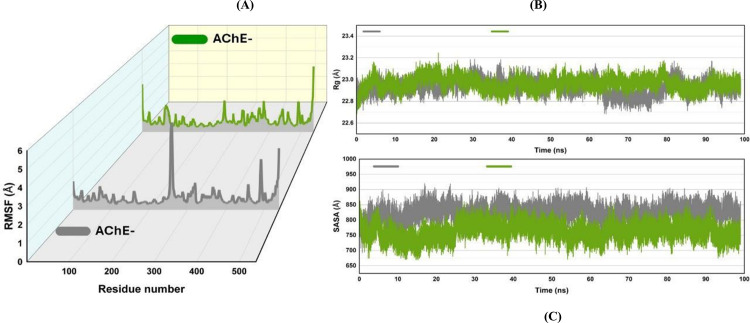
RMSF (A) which indicates the flexibility of the complexes, Radius of Gyration (B) which describes the compactness of the protein, and SASA (C) which reflects the area of the protein that was exposed for the interaction of solvent for the analysis of CCL and AZ-29 bound AChE complexes in gray and green color respectively.

Fig 11 (A) provides the root mean square fluctuation (RMSF) plot to describe the protein residues’ movement during the entire simulation. In this plot, the gray line is the RMSF of the CCL-bound AChE and the green line is the RMSF of the AZ-29 bound AChE. Subsequent analysis concludes that regions with higher RMSF values are present towards the center and around residue number 300. The end of the protein looks more flexible.

### Radius of gyration (Rg) plot

Fig 11(B) shows the radius of gyration (Rg) for Acetylcholinesterase (AChE) during the 100 ns simulation that describes the structural stability as well as the compactness of the protein throughout the MD simulation. In this plot, the gray color shows the CCL-bound AChE and the green color represents the AZ-29 ligand-bound AChE. The Rg values vary in the range of 22.6 –23.4 Å demonstrating that the protein size remains quite invariant. However, the remainder of the rationality of the structure is convincingly obvious: AChE does not unfold the protein, and remains compact. During the first quarter of the simulation, there is a mild fluctuation that triggers the equilibration of the protein; nonetheless, the curve becomes stable with an average Rg of 23.0–23.2 Å.

### Solvent accessible surface area (SASA) plot (c)

The graphical representation of the solvent accessible surface area (SASA) in Fig 11(C) for AChE for a period of 100 ns offers an understanding of how much area of the protein is exposed to the solvent during the molecular dynamic study.

According to this graph, SASA is oscillating in the range of 650–950 Å² while the most probable density is roughly at 750–850 Å². These values vary mostly in the constant range which indicates that AChE keeps a relatively constant level of the solvent surface exposure. However, since there are no large, sustained deviations in SASA, this implies that AChE does not undergo large-scale unfolding or changing of structures throughout the simulation.

**Fig 12:**
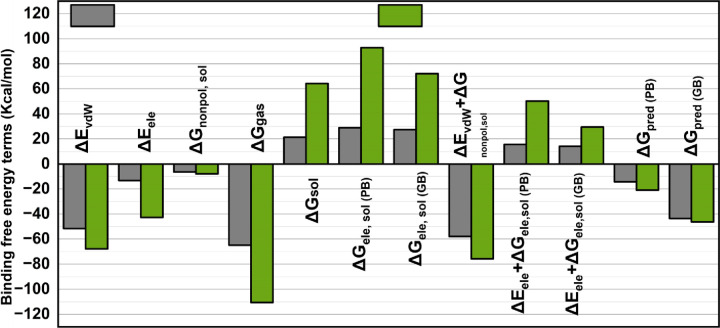
Decomposition of Binding Free Energy Terms: comparison of Energy Contributions between CCL (gray) and AZ-29 (green) using Poisson-Boltzmann (PB) and Generalized Born (GB) Models.

Fig 12 illustrates the decomposition of binding free energy into various contributing terms, with the x-axis representing different energy components and the y-axis showing their corresponding values in kcal/mol. The green (AZ-29) and gray (CCL) bars indicate results from different computational methods, distinguishing favorable energy contributions (negative values) from unfavorable ones (positive values). Key energy components in binding free energy calculations include van der Waals energy, electrostatic energy, non-polar solvation free energy, gas-phase energy, and total solvation free energy, with electrostatic solvation assessed using the Poisson-Boltzmann (PB) and Generalized Born (GB) models. The PB approach is computationally intensive with detailed calculations, whereas the GB approach is a lower energy density but faster (though possibly less accurate) approximation of the electrostatic interactions.

The bars in the figure represent the difference in the predicted binding affinity of ΔG-pred (PB) and ΔG-pred (GB), with the difference in binding (negative value) of ΔG-pred (GB) looking a lot stronger than that of ΔG-pred (PB). Such differences indicate the variations in the approximations of all electrostatic contributions and solvation effects by both methods, which provide an understanding of the relative accuracy and reliability of both PB and GB to predict binding affinities in molecular systems.

## Discussion

Alzheimer’s disease is a prevalent and serious neurodegenerative condition. AD progression is driven by a combination of cholinergic deficits, amyloid-β accumulation, tau hyperphosphorylation, and inflammatory responses, which together lead to synaptic loss and cognitive decline [5]. Unfortunately, the existing medications for managing Alzheimer’s have proven to be less effective, leading to a strong push for the development of new and innovative treatment strategies [29]. Natural products are the source of lead molecules, and many synthetic drugs are developed based on details from natural products [30]. Many scientific studies have proven that several medicinal plants and their primary phytochemicals can treat AD [31]. We explored 40 possible phytochemicals from *Astragalus zederbaueri* using an *in silico* approach to identify the most effective inhibitors for AD treatment. A combination of bioactive molecules like saponins, polysaccharides, flavonoids, and alkaloids characterize *Astragalus zederbaueri* [32]. Acetylcholinesterase (AChE) is an enzyme that decomposes Acetylcholine, resulting in reduced neurotransmission and progressive cognitive impairment. This coincides with previous studies that indicate that plant-based compounds are capable of affecting AD targets such as cholinesterase enzymes, oxidative stress responses, and tau phosphorylation signaling [6]. Inhibition of AChE can enhance cholinergic transmission, which suppresses the AD symptoms that include loss of memory, as well as the possibility of reducing increased mortality that may come along with AD [33]. AChE regulates peptide aggregation and the formation of amyloid-beta. Moreover, tau-associated kinases, including TTBK1 and TTBK2, have also been reported to facilitate tau aggregation, and computational techniques indicate that the imbalance of these kinases disrupts tau-related processes, which also play a role in the pathology of AD [34]. To confirm the crystal structure of AChE found in the Protein Data Bank, a Ramachandran plot was created that indicated that 90.3% of the residues were found in favored regions. The docking outcome showed that Rutin (AZ-29) exhibited the highest binding affinity following co-crystal ligand donepezil (CCL), with a result of −15.043; AZ-24 showed the lowest binding affinity of −0.812. CCL and AZ-29 had extensive hydrogen-bonded and stacked π-π binding interactions with key active site residues, which provide a structural rationale for prioritizing these compounds for downstream experimental testing as AChE inhibitors. CCL shows several positive pharmacokinetic characteristics, but rather, AZ-29, AZ-32, and AZ-28 show favorable *in silico* docking/MD behavior and predicted ADMET features, supporting their prioritization as computational lead candidates pending experimental confirmation. Of special importance is AZ-29, which is more widely distributed, possesses a greater unbound fraction, and is not hepatotoxic. The analysis of these compounds, done by using the density functional theory (DFT) explained their electronic characteristics, and CCL and AZ-29 possess smaller HOMO-LUMO gaps, thus becoming more reactive and capable of binding to biological systems in accordance with the docking experiments. The molecular dynamics (MD) simulation is used to give vital information about the interactions and stability of Acetylcholinesterase (AChE) with ligands CCL and AZ-29. RMSD trends are consistent with a stable complex over the simulated window, with profiles remaining within a narrow range after equilibration, suggesting that ligand binding does not induce major structural drift and that the binding pocket remains stable; however, longer simulations and replicate runs would be required to assess convergence fully. Stability may be enhanced in specific parts of the proteins by ligand binding; AZ-29 and CCL are both stabilized. Moreover, the radius of gyration (Rg) means that AChE has a compact conformation and reduced conformational change; the solvent-accessible surface area (SASA) values imply that it is always in contact with solvent. The binding free energy decomposition has shown that AZ-29 binds with a higher affinity than CCL, which was probably because it interacts well with residues in the active site.

The primary drawback of molecular docking is the omission of explicit solvent and structural waters which are essential in precise prediction of protein-ligand binding. Also, docking in practice is generally single-target, neglecting multi-target interactions, including those of butyrylcholinesterase (BChE) or tau-related proteins in Alzheimer, and it is hard to determine the full efficacy of any compound in more complex biological systems.

## Conclusion

In this study *in silico* examination performed on the isolated phytochemicals of *Astragalus zederbaueri*, specifically Rutin (AZ-29), Kaempferol-3-O-rutinoside (AZ-32), and Isoquercitrin (AZ-28), displays great AChE inhibitory capacity in treating Alzheimer’s disease. Rutin exhibits the greatest binding affinity and stable interactions with binding residues TRP341 and TYR286. The molecular dynamics simulations suggested stable ligand-enzyme complexes. The toxicological and pharmacokinetic studies also revealed that these compounds are safe. Our findings indicate that Rutin and other similar compounds might make good therapeutic candidates for AChE-target therapies and offer a potential path forward in the treatment of AD through enhancing cholinergic activity.

## Supporting information

S1 Table Compounds codes, names, 2D, 3D and Smiles. (DOCX)

S2 Table Compounds with PubChem CID and Retrieval Date. (DOCX)

S3 Table pkCSM pharmacokinetic parameters of the selected phytochemicals from *Astragalus zederbaueri* having the highest inhibitory effects (DOCX)

S1 Fig Heat map of docking scores for AChE with 40 phytocompounds and CCL. (DOCX)

S2 Fig The heatmap of molecular properties highlights key attributes of the studied compounds. (DOCX)

S2 File Graphical Abstract. (TIF)
