# Supplementary material for: Phytochemicals from Astragalus zederbaueri as Acetylcholinesterase Inhibitors for Alzheimer’s Therapy
Source: PLoS One. 2026 Apr 10;21(4):e0346177. doi: 10.1371/journal.pone.0346177 (PMC13068338; doi:10.1371/journal.pone.0346177)
Supplement: S1 Table — (DOCX) [file pone.0346177.s001.docx]

**Supplementary Table S1.** Compounds codes, names, 2D, 3D and Smiles

| **Code** | **Compound Name** | **2D Structure** | **3 D Structure** | **SMILES** |
| --- | --- | --- | --- | --- |
| AZ-1 | Quinic acid |  | 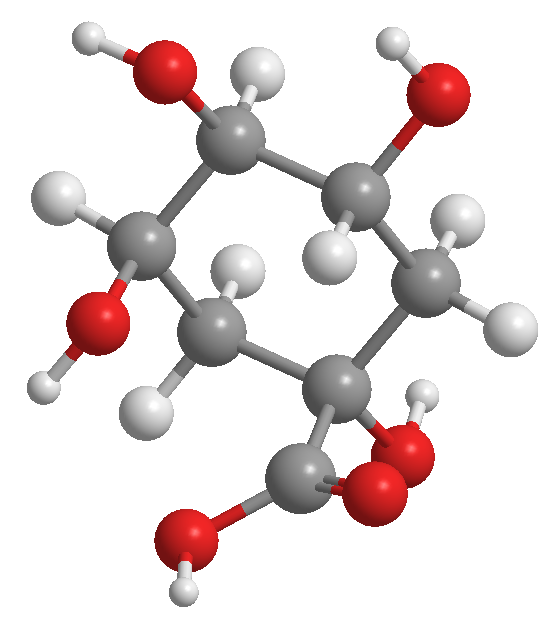 | OC1(C(O)=O)C[C@@H](O)C(O)[C@H](O)C1 |
| AZ-2 | Trigonelline |  | 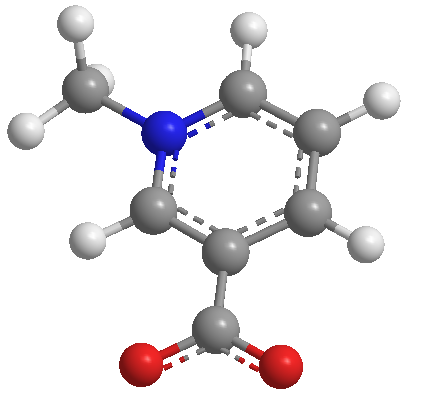 | O=C([O-])C1=C[N+](C)=CC=C1 |
| AZ-3 | Citric acid |  | 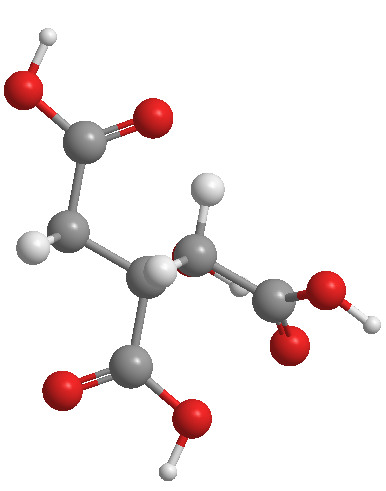 | O=C(O[H])C(O[H])(CC(O[H])=O)CC(O[H])=O |
| AZ-4 | Gallic acid (3,4,5-Trihydroxybenzoic acid) |  | 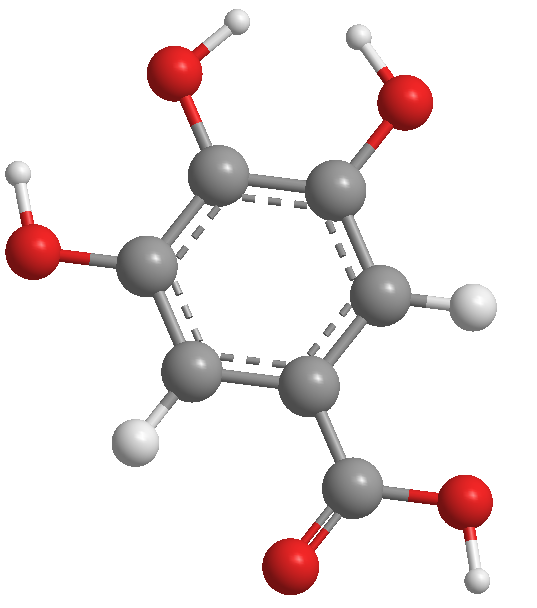 | O=C(O[H])C1=CC(O[H])=C(O[H])C(O[H])=C1 |
| AZ-5 | Tryptamine |  | 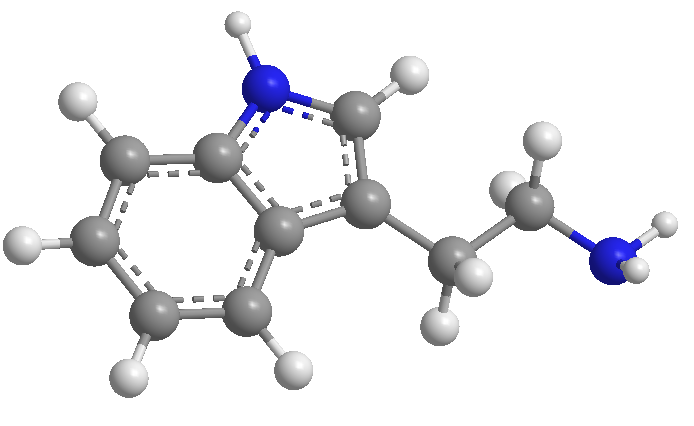 | [H]N1C2=CC=CC=C2C(CCN([H])[H])=C1 |
| AZ-6 | Dihydroxybenzoic acid |  | 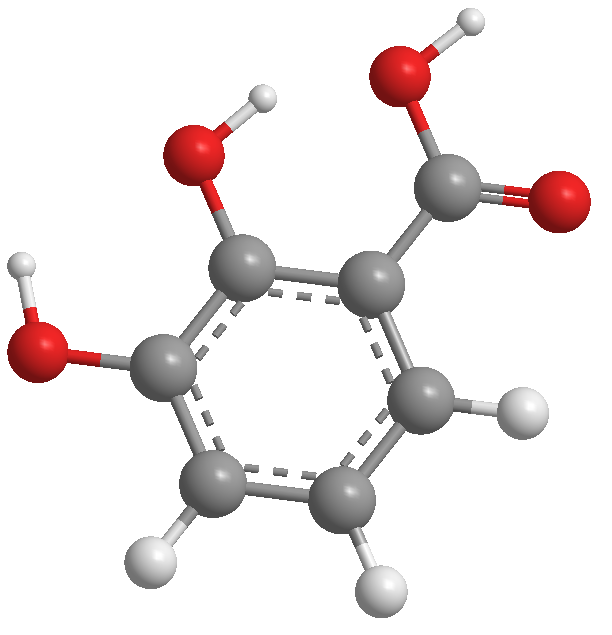 | O=C(O[H])C1=C(O[H])C(O[H])=CC=C1 |
| AZ-7 | Pantothenic acid |  | 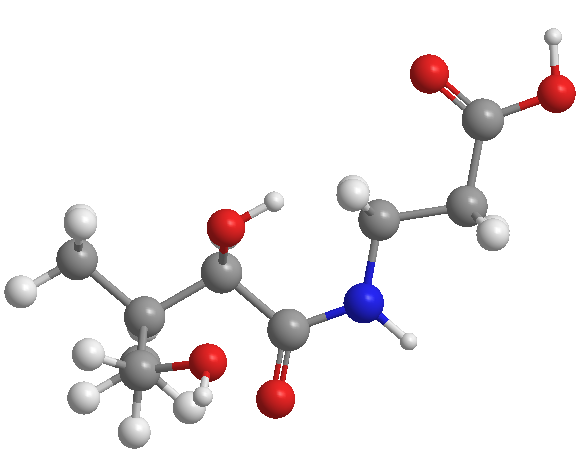 | CC(C)(CO)[C@@H](O)C(N(CCC(O)=O)[H])=O |
| AZ-8 | Salidroside |  | 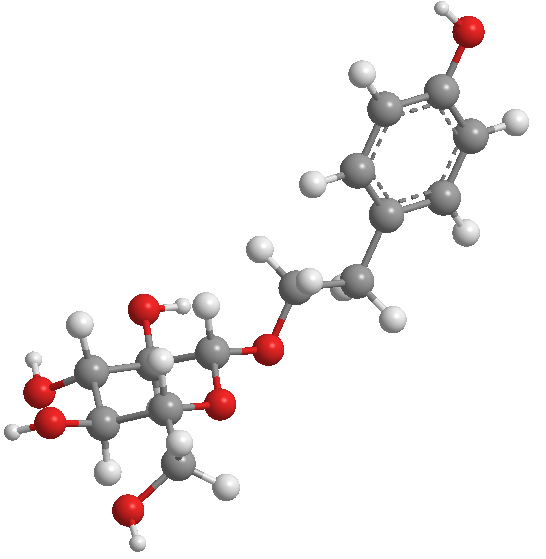 | [H]OC1=CC=C(CCO[C@H]2[C@H](O[H])[C@@H](O[H])[C@H](O[H])[C@@H](CO[H])O2)C=C1 |
| AZ-9 | Uralenneoside |  | 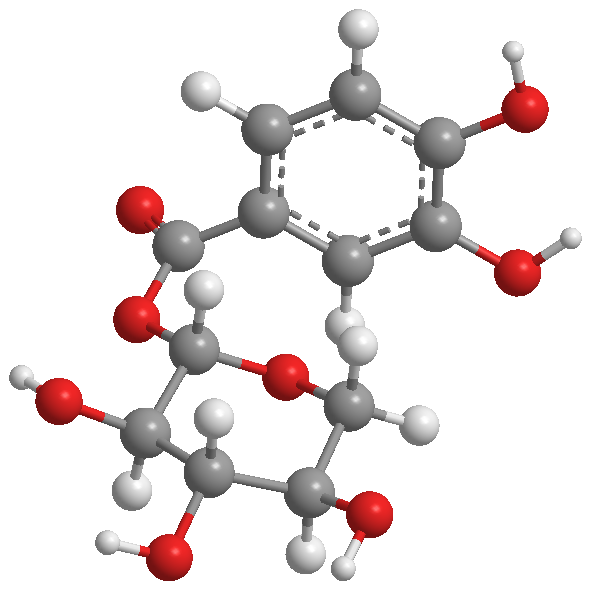 | O=C(C1=CC(O[H])=C(O[H])C=C1)O[C@@H]2OC[C@@H](O[H])[C@H](O[H])[C@H]2O[H] |
| AZ-10 | Hydroxypimelic acid |  | 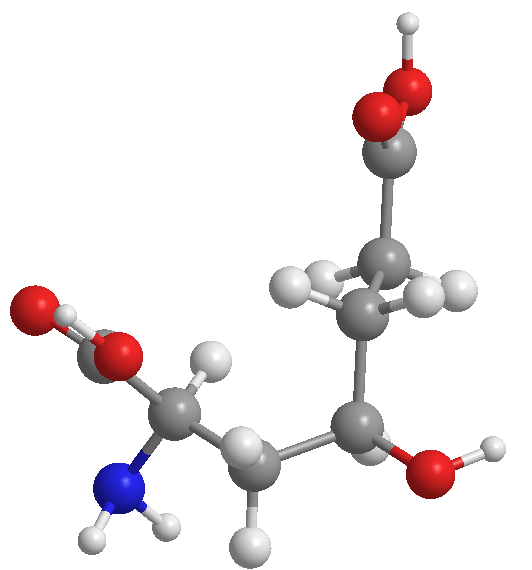 | O=C(C(N([H])[H])CC(O[H])CCC(O[H])=O)O[H] |
| AZ-11 | Coumaroylquinic acid |  | 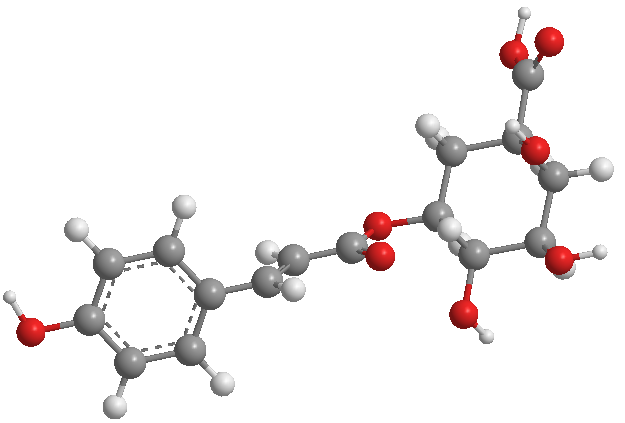 | O=C(O[C@@H]1C[C@](O[H])(C(O[H])=O)C[C@@H](O)[C@H]1O)/C([H])=C([H])/C2=CC=C(O[H])C=C2 |
| AZ-12 | Coumaric acid hexoside |  | 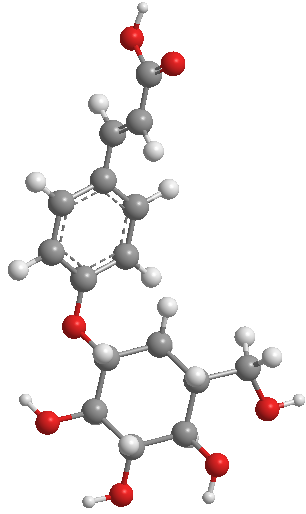 | OCC1C(O)C(O)C(O)C(OC2=CC=C(/C([H])=C([H])/C(O)=O)C=C2)C1 |
| AZ-13 | Caffeic acid hexoside |  | 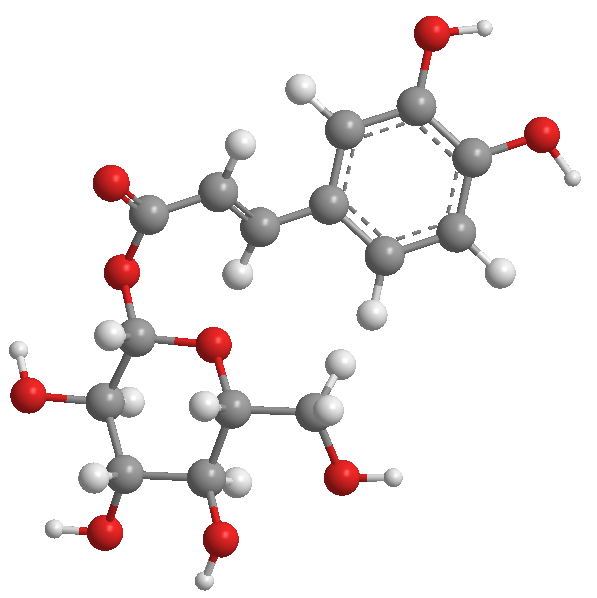 | OCC1C(O)C(O)C(O)C(OC(/C([H])=C([H])/C2=CC(O)=C(O)C=C2)=O)O1 |
| AZ-14 | Osmanthuside H |  | 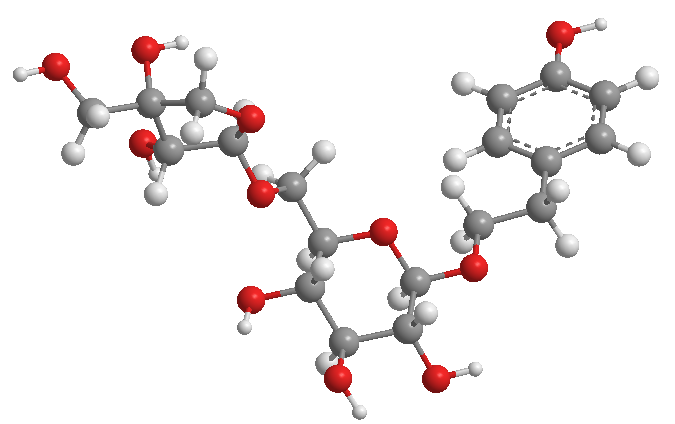 | OC1=CC=C(CCO[C@H]2[C@H](O)[C@@H](O)[C@H](O)[C@@H](CO[C@@H]3OC[C@](O)(CO)[C@H]3O)O2)C=C1 |
| AZ-15 | 3-O-Feruloylquinic acid |  | 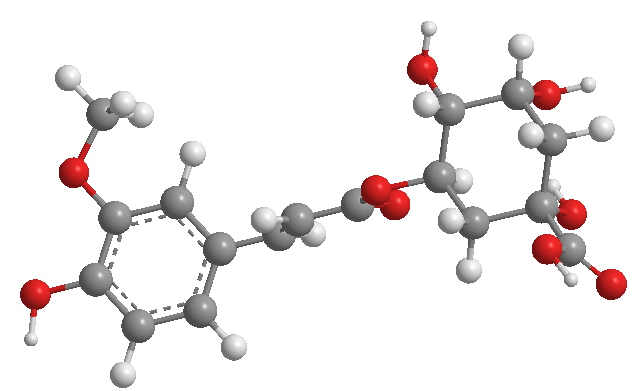 | OC1=C(OC)C=C(/C([H])=C([H])/C(O[C@@H]2C[C@](O)(C(O)=O)C[C@@H](O)[C@H]2O)=O)C=C1 |
| AZ-16 | Pimelic acid |  | 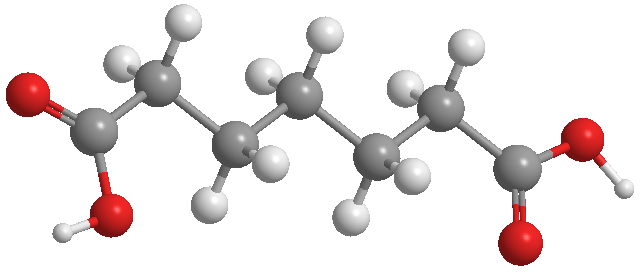 | OC(CCCCCC(O)=O)=O |
| AZ-17 | Caffeic acid |  | 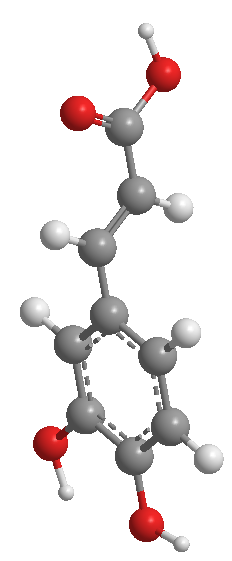 | OC1=C(O)C=CC(/C([H])=C(C(O)=O)\[H])=C1 |
| AZ-18 | Ferulic acid |  | 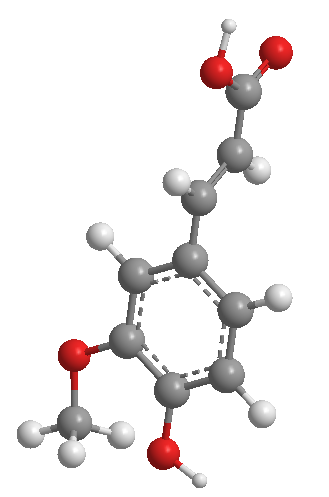 | OC1=C(OC)C=C(/C([H])=C(C(O)=O)\[H])C=C1 |
| AZ-19 | Kynurenic acid |  | 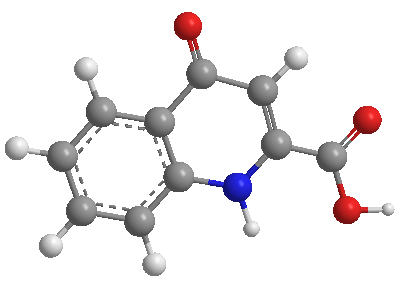 | O=C1C=C(C(O)=O)N([H])C2=CC=CC=C21 |
| AZ-20 | Tuberonic acid |  | 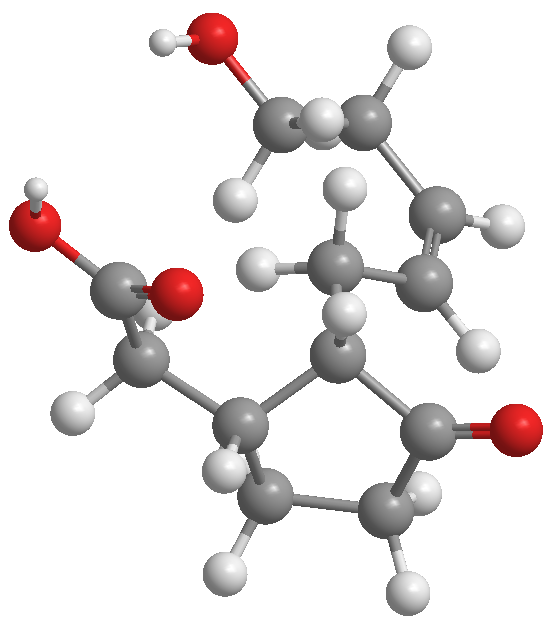 | OC(C[C@H]1CCC([C@H]1C/C([H])=C([H])\CCO)=O)=O |
| AZ-21 | Riboflavin |  | 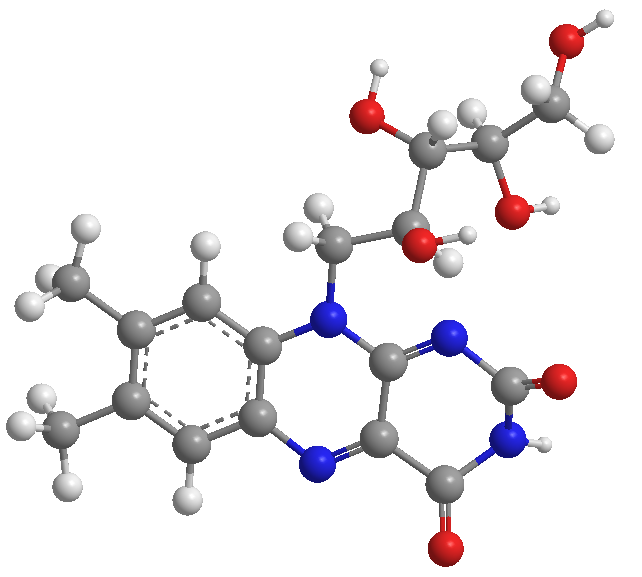 | CC1=CC2=C(N=C(C(N3[H])=O)C(N2C[C@H](O[H])[C@H](O)[C@H](O)CO)=NC3=O)C=C1C |
| AZ-22 | 5-O-Feruloylquinic acid |  | 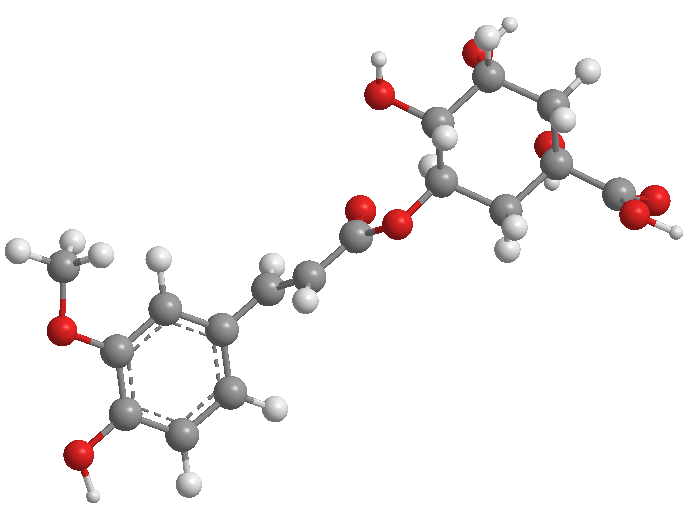 | OC1=C(OC)C=C(/C([H])=C([H])/C(O[C@@H]2C[C@](O[H])(C(O)=O)C[C@@H](O[H])[C@H]2O[H])=O)C=C1 |
| AZ-23 | *p*-Coumaric acid |  | 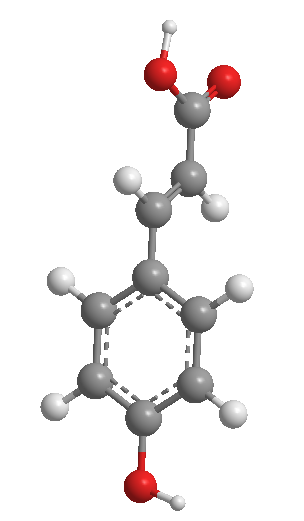 | OC1=CC=C(/C([H])=C(C(O)=O)\[H])C=C1 |
| AZ-24 | 3-Hydroxysuberic acid |  | 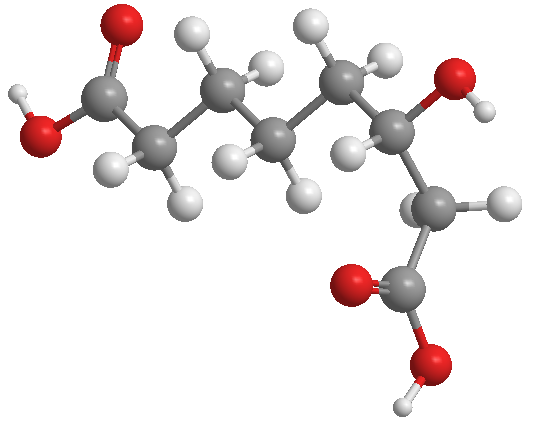 | OC(CCCCC(O)CC(O)=O)=O |
| AZ-25 | 4-O-Feruloylquinic acid |  | 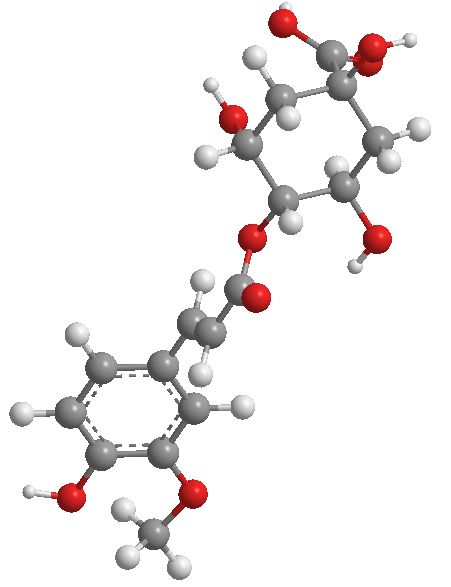 | OC1=C(OC)C=C(/C([H])=C(C(OC2[C@H](O)CC(O[H])(C(O)=O)C[C@H]2O)=O)\[H])C=C1 |
| AZ-26 | Quercetin 3-O-alpha-rhamnopyranoside |  | 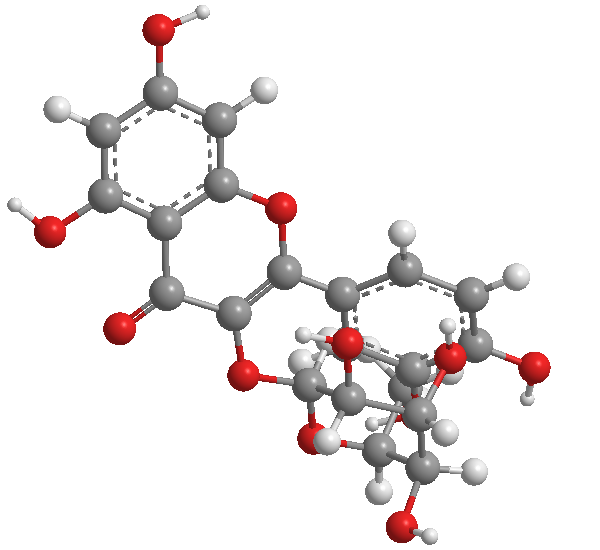 | C[C@H]1[C@@H]([C@H]([C@H]([C@@H](O1)OC2=C(OC3=CC(=CC(=C3C2=O)O)O)C4=CC(=C(C=C4)O)O)O)O)O |
| AZ-27 | Benzoylmalic acid |  | 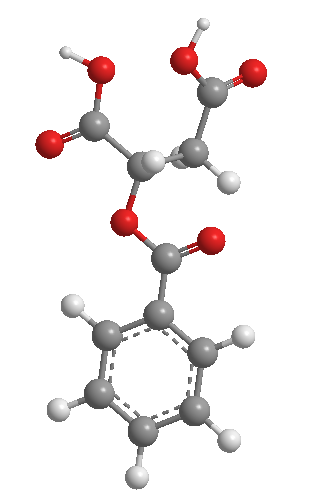 | O=C(OC(C(O)=O)([H])CC(O)=O)C1=CC=CC=C1 |
| AZ-28 | Isoquercitrin |  | 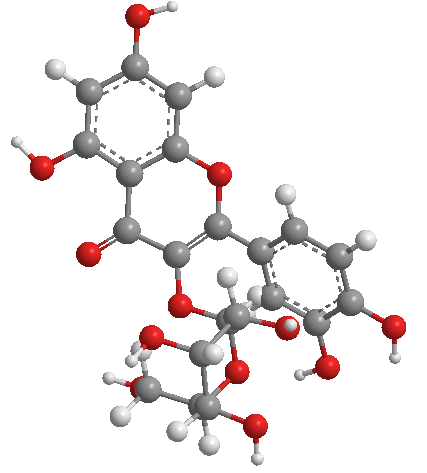 | OC1=CC(O)=C(C(C(O[C@@H]2O[C@H](CO)[C@@H](O)[C@H](O)[C@H]2O)=C(C3=CC(O)=C(O)C=C3)O4)=O)C4=C1 |
| AZ-29 | Rutin (Quercetin-3-O-rutinoside) |  | 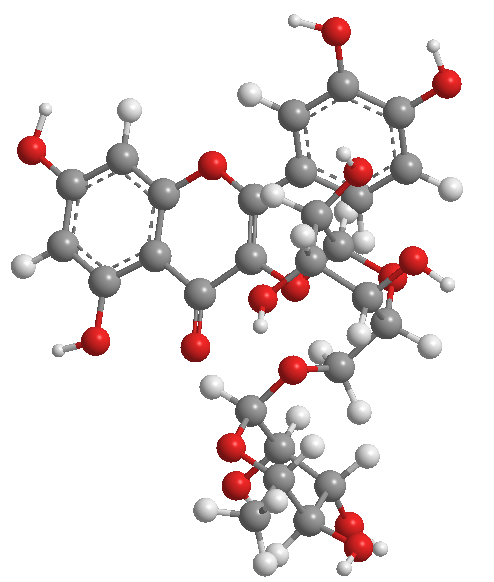 | OC1=C(O)C=CC(C(OC2=CC(O)=CC(O)=C23)=C(O[C@@H]4O[C@H](CO[C@@H]5O[C@@H](C)[C@H](O)[C@@H](O)[C@H]5O)[C@@H](O)[C@H](O)[C@H]4O)C3=O)=C1 |
| AZ-30 | Azelaic acid (Nonanedioic acid) |  | 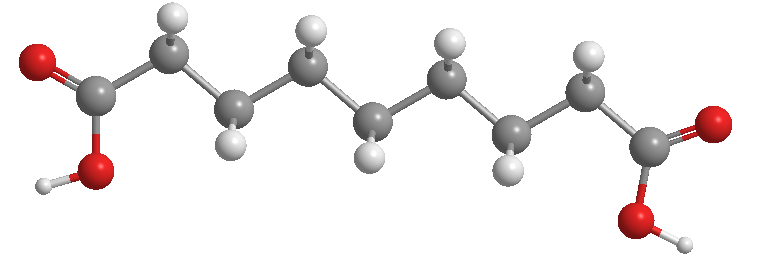 | OC(CCCCCCCC(O)=O)=O |
| AZ-31 | Astragalin (Kaempferol-3-O-glucoside) |  | 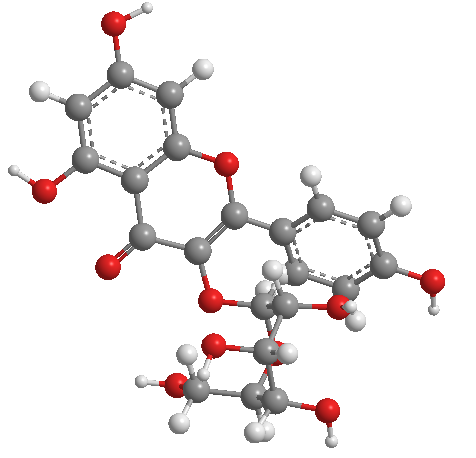 | OC1=CC(O)=C(C(C(O[C@@H]2O[C@H](CO)[C@@H](O)[C@H](O)[C@H]2O)=C(C3=CC=C(O)C=C3)O4)=O)C4=C1 |
| AZ-32 | Kaempferol-3-O-rutinoside (Nicotiflorin) |  | 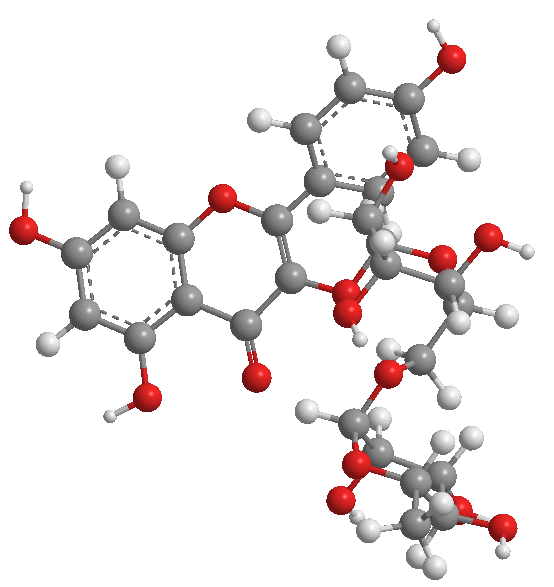 | OC1=C(O)C=CC(C(OC2=CC(O)=CC(O)=C23)=C(O[C@@H]4O[C@H](CO[C@@H]5O[C@@H](C)[C@H](O)[C@@H](O)[C@H]5O)[C@@H](O)[C@H](O)[C@H]4O)C3=O)=C1 |
| AZ-33 | Naringenin (4′,5,7-Trihydroxyflavanone) |  | 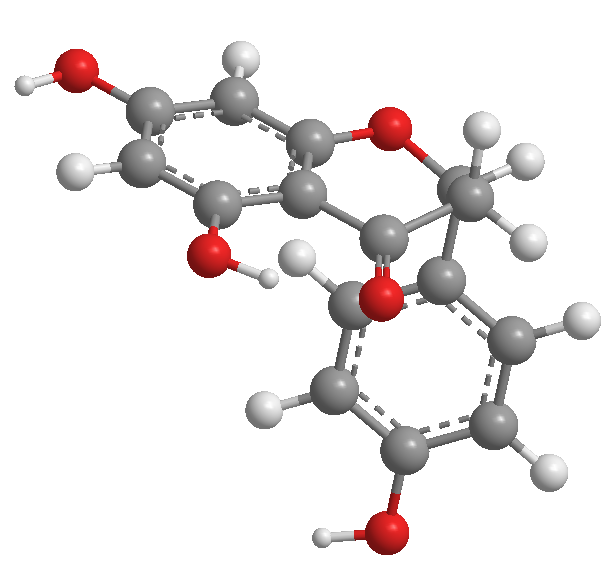 | OC1=C2C(O[C@H](C3=CC=C(O)C=C3)CC2=O)=CC(O)=C1 |
| AZ-34 | Jasmonic acid |  | 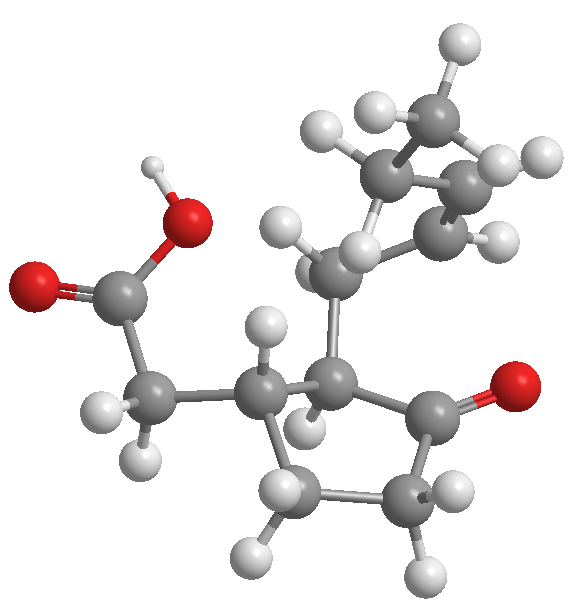 | O=C1CC[C@H](CC(O)=O)[C@H]1C/C([H])=C(CC)/[H] |
| AZ-35 | Quercetin (3,3′,4′,5,7-Pentahydroxyflavone) |  | 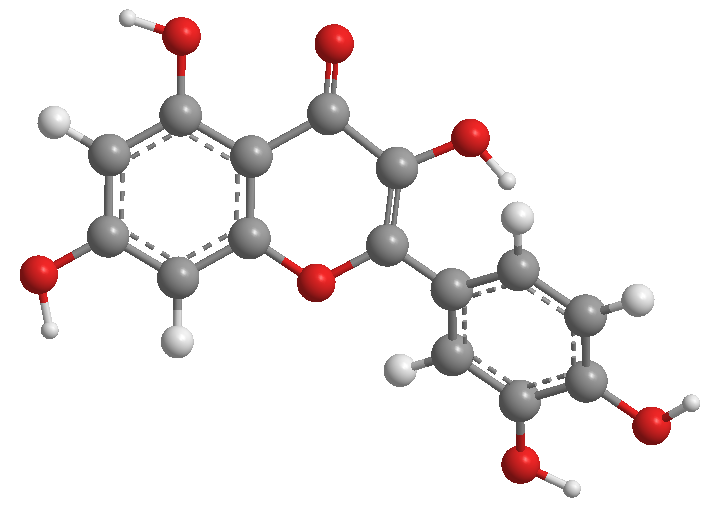 | OC1=C2C(OC(C3=CC(O)=C(C=C3)O)=C(O)C2=O)=CC(O)=C1 |
| AZ-36 | Sebacic acid (Decanedioic acid) |  | 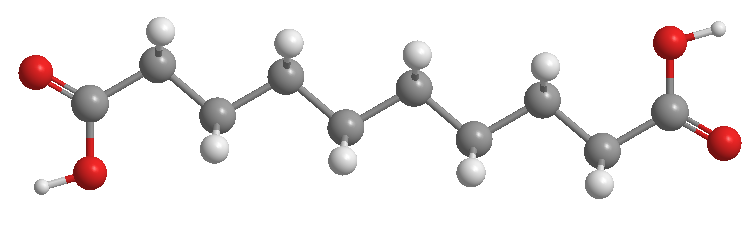 | OC(CCCCCCCCC(O)=O)=O |
| AZ-37 | Luteolin (3′,4′,5,7-Tetrahydroxyflavone) |  | 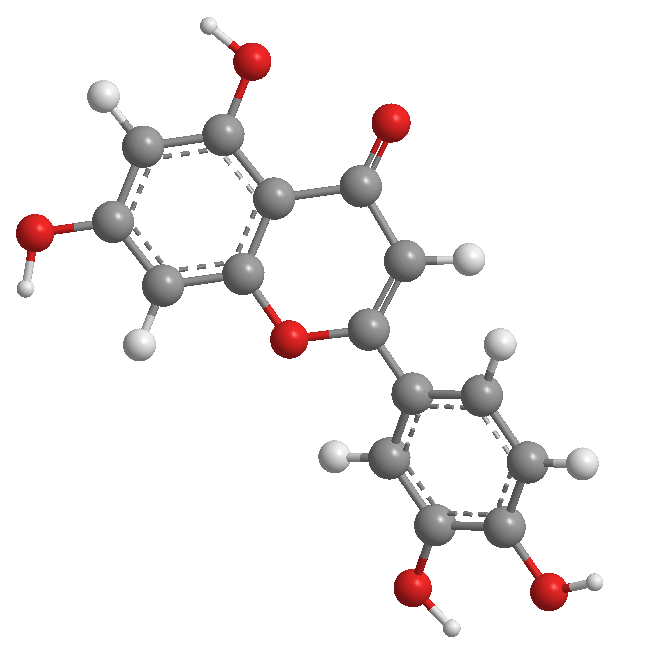 | OC1=C2C(OC(C3=CC(O)=C(C=C3)O)=CC2=O)=CC(O)=C1 |
| AZ-38 | Kaempferol (3,4′,5,7-Tetrahydroxyflavone) |  | 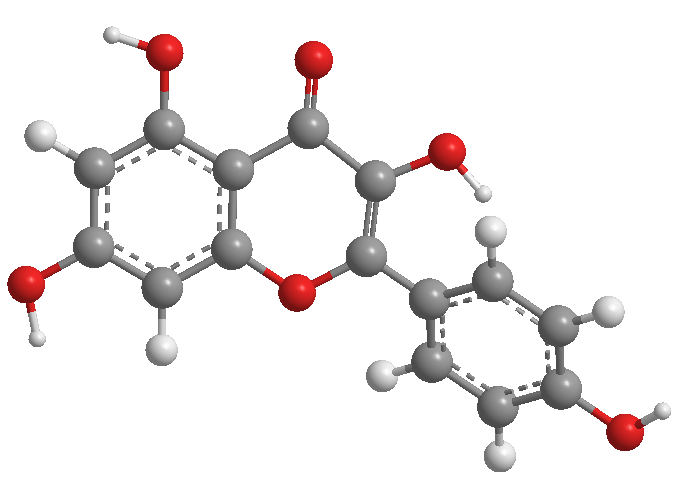 | OC1=C2C(OC(C3=CC=C(C=C3)O)=C(O)C2=O)=CC(O)=C1 |
| AZ-39 | Apigenin (4′,5,7-Trihydroxyflavone) |  | 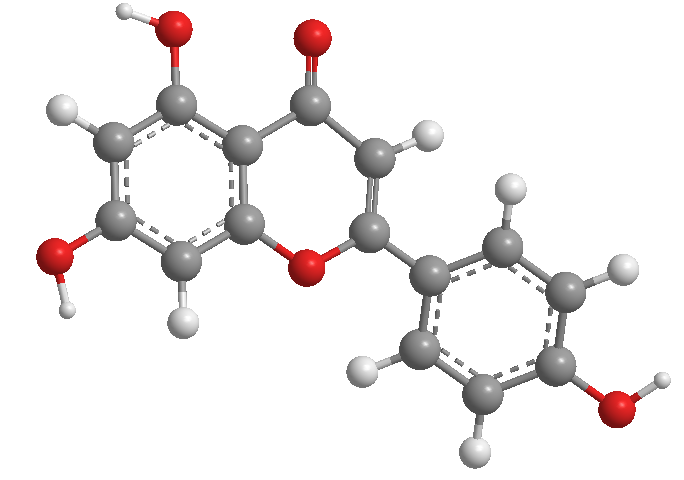 | OC1=C2C(OC(C3=CC=C(C=C3)O)=CC2=O)=CC(O)=C1 |
| AZ-40 | Isoliquiritigenin |  | 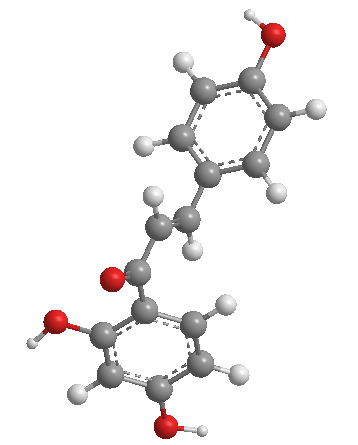 | OC1=CC=C(/C([H])=C([H])/C(C2=C(O)C=C(O)C=C2)=O)C=C1 |
