# Supplementary material for: Phytochemicals from Astragalus zederbaueri as Acetylcholinesterase Inhibitors for Alzheimer’s Therapy
Source: PLoS One. 2026 Apr 10;21(4):e0346177. doi: 10.1371/journal.pone.0346177 (PMC13068338; doi:10.1371/journal.pone.0346177)
Supplement: S2 Table — (DOCX) [file pone.0346177.s002.docx]

**Supplementary Table S2.** Compounds with PubChem CID and Retrieval Date

| **Code** | **Compound Name** | **PubChem CID** | **Retrieval Date** |
| --- | --- | --- | --- |
| AZ-1 | Quinic acid | 37439 | 15-07-2024 |
| AZ-2 | Trigonelline | 5570 | 15-07-2024 |
| AZ-3 | Citric acid | 311 | 15-07-2024 |
| AZ-4 | Gallic acid (3,4,5-Trihydroxybenzoic acid) | 6476954 | 15-07-2024 |
| AZ-5 | Tryptamine | 1150 | 15-07-2024 |
| AZ-6 | Dihydroxybenzoic acid | 19 | 15-07-2024 |
| AZ-7 | Pantothenic acid | 6613 | 15-07-2024 |
| AZ-8 | Salidroside | 159278 | 15-07-2024 |
| AZ-9 | Uralenneoside | 132594 | 15-07-2024 |
| AZ-10 | Hydroxypimelic acid | 54514417 | 15-07-2024 |
| AZ-11 | Coumaroylquinic acid | 6441280 | 15-07-2024 |
| AZ-12 | Coumaric acid hexoside | 13783633 | 15-07-2024 |
| AZ-13 | Caffeic acid hexoside | 6124135 | 15-07-2024 |
| AZ-14 | Osmanthuside H | 192437 | 15-07-2024 |
| AZ-15 | 3-O-Feruloylquinic acid | 9799386 | 15-07-2024 |
| AZ-16 | Pimelic acid | 385 | 15-07-2024 |
| AZ-17 | Caffeic acid | 689043 | 15-07-2024 |
| AZ-18 | Ferulic acid | 445858 | 15-07-2024 |
| AZ-19 | Kynurenic acid | 3845 | 15-07-2024 |
| AZ-20 | Tuberonic acid | 6443968 | 15-07-2024 |
| AZ-21 | Riboflavin | 493570 | 15-07-2024 |
| AZ-22 | 5-O-Feruloylquinic acid | 10133609 | 15-07-2024 |
| AZ-23 | *p*-Coumaric acid | 637542 | 15-07-2024 |
| AZ-24 | 3-Hydroxysuberic acid | 22328017 | 15-07-2024 |
| AZ-25 | 4-O-Feruloylquinic acid | 10177048 | 15-07-2024 |
| AZ-26 | Quercetin 3-O-alpha-rhamnopyranoside | 5280459 | 15-07-2024 |
| AZ-27 | Benzoylmalic acid | 13953341 | 15-07-2024 |
| AZ-28 | Isoquercitrin | 5484006 | 15-07-2024 |
| AZ-29 | Rutin (Quercetin-3-O-rutinoside) | 5280805 | 15-07-2024 |
| AZ-30 | Azelaic acid (Nonanedioic acid) | 23615575 | 15-07-2024 |
| AZ-31 | Astragalin (Kaempferol-3-O-glucoside) | 5282102 | 15-07-2024 |
| AZ-32 | Kaempferol-3-O-rutinoside (Nicotiflorin) | 5318767 | 15-07-2024 |
| AZ-33 | Naringenin (4′,5,7-Trihydroxyflavanone) | 932 | 15-07-2024 |
| AZ-34 | Jasmonic acid | 5281166 | 15-07-2024 |
| AZ-35 | Quercetin (3,3′,4′,5,7-Pentahydroxyflavone) | 5280343 | 15-07-2024 |
| AZ-36 | Sebacic acid (Decanedioic acid) | 86609903 | 15-07-2024 |
| AZ-37 | Luteolin (3′,4′,5,7-Tetrahydroxyflavone) | 15661823 | 15-07-2024 |
| AZ-38 | Kaempferol (3,4′,5,7-Tetrahydroxyflavone) | 86278401 | 15-07-2024 |
| AZ-39 | Apigenin (4′,5,7-Trihydroxyflavone) | 5280443 | 15-07-2024 |
| AZ-40 | Isoliquiritigenin | 638278 | 15-07-2024 |
