# Supplementary material for: Phytochemicals from Astragalus zederbaueri as Acetylcholinesterase Inhibitors for Alzheimer’s Therapy
Source: PLoS One. 2026 Apr 10;21(4):e0346177. doi: 10.1371/journal.pone.0346177 (PMC13068338; doi:10.1371/journal.pone.0346177)
Supplement: S3 Table — (DOCX) [file pone.0346177.s003.docx]

**Supplementary Table S3: pkCSM pharmacokinetic parameters of the selected phytochemicals from *Astragalus zederbaueri* having the highest inhibitory effects**

| **Pharmacokinetic Properties** | | **Selected Phytochemicals** | | | |
| --- | --- | --- | --- | --- | --- |
| **Properties** | **Model Name** | **CCL** | **AZ-29** | **AZ-32** | **AZ-28** |
| **Absorption** | **Water solubility** | -4.648 | -2.892 | -2.88 | -3.09 |
|  | **Caco2 permeability** | 1.273 | -0.949 | -0.774 | 0.052 |
|  | **Intestinal absorption (human)** | 93.707 | 23.446 | 20.727 | 33.509 |
|  | **Skin Permeability** | -2.585 | -2.735 | -2.735 | -2.735 |
|  | **P-glycoprotein substrate** | Yes | Yes | Yes | Yes |
|  | **P-glycoprotein I inhibitor** | Yes | No | No | No |
|  | **P-glycoprotein II inhibitor** | Yes | No | No | No |
| **Distribution** | **VDss (human)** | 1.266 | 1.663 | 0.07 | -0.188 |
|  | **Fraction unbound (human)** | 0 | 0.187 | 0.282 | 0.156 |
|  | **BBB permeability** | 0.157 | -1.899 | -2.213 | -1.887 |
|  | **CNS permeability** | -1.464 | -5.178 | -5.439 | -5.034 |
| **Metabolism** | **CYP2D6 substrate** | Yes | No | No | No |
|  | **CYP3A4 substrate** | Yes | No | No | No |
|  | **CYP1A2 inhibitor** | No | No | No | No |
|  | **CYP2C19 inhibitor** | No | No | No | No |
|  | **CYP2C9 inhibitor** | No | No | No | No |
|  | **CYP2D6 inhibitor** | Yes | No | No | No |
|  | **CYP3A4 inhibitor** | Yes | No | No | No |
| **Excretion** | **Total Clearance** | 0.987 | -0.369 | -0.138 | 0.715 |
|  | **Renal OCT2 substrate** | Yes | No | No | No |
| **Toxicity** | **AMES toxicity** | No | No | Yes | Yes |
|  | **Max. tolerated dose (human)** | -0.217 | 0.452 | 0.422 | 1.12 |
|  | **hERG I inhibitor** | No | No | No | No |
|  | **hERG II inhibitor** | Yes | Yes | Yes | Yes |
|  | **Oral Rat Acute Toxicity (LD50)** | 2.753 | 2.491 | 2.348 | 2.922 |
|  | **Oral Rat Chronic Toxicity (LOAEL)** | 0.991 | 3.673 | 5.32 | 3.926 |
|  | **Hepatotoxicity** | Yes | No | No | No |
|  | **Skin Sensitization** | No | No | No | No |
|  | ***T.Pyriformis* toxicity** | 0.804 | 0.285 | 0.285 | 0.285 |
|  | **Minnow toxicity** | -2.011 | 7.677 | 7.091 | 5.994 |
