# Supplementary figures and images for: Phytochemicals from Astragalus zederbaueri as Acetylcholinesterase Inhibitors for Alzheimer’s Therapy
Source: PLoS One. 2026 Apr 10;21(4):e0346177. doi: 10.1371/journal.pone.0346177 (PMC13068338; doi:10.1371/journal.pone.0346177)

**Supplementary Figure S1.** Heat map of docking scores for AChE with 40 phytocompounds and CCL


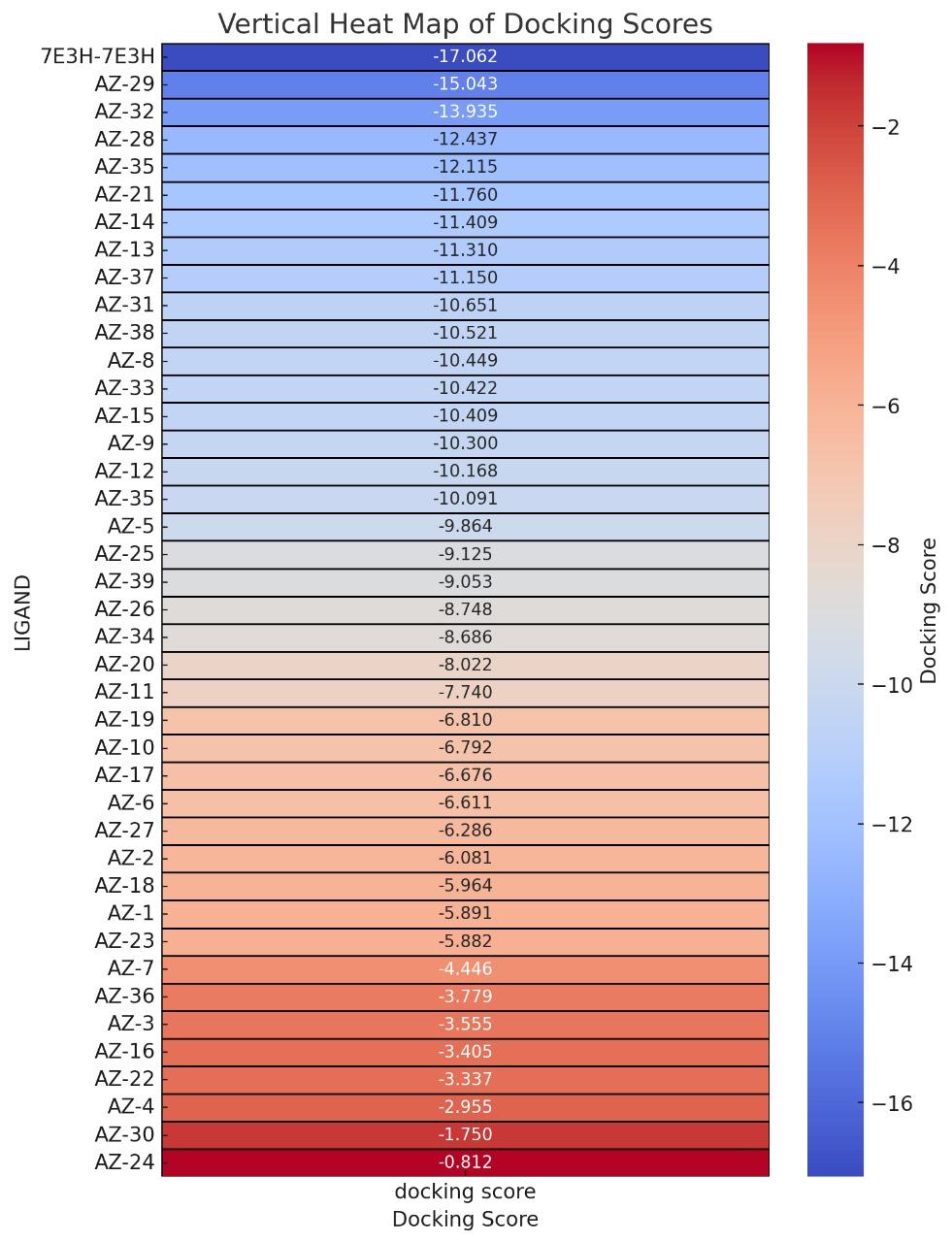

Supplement: S1 Fig — (DOCX) [file pone.0346177.s004.docx]

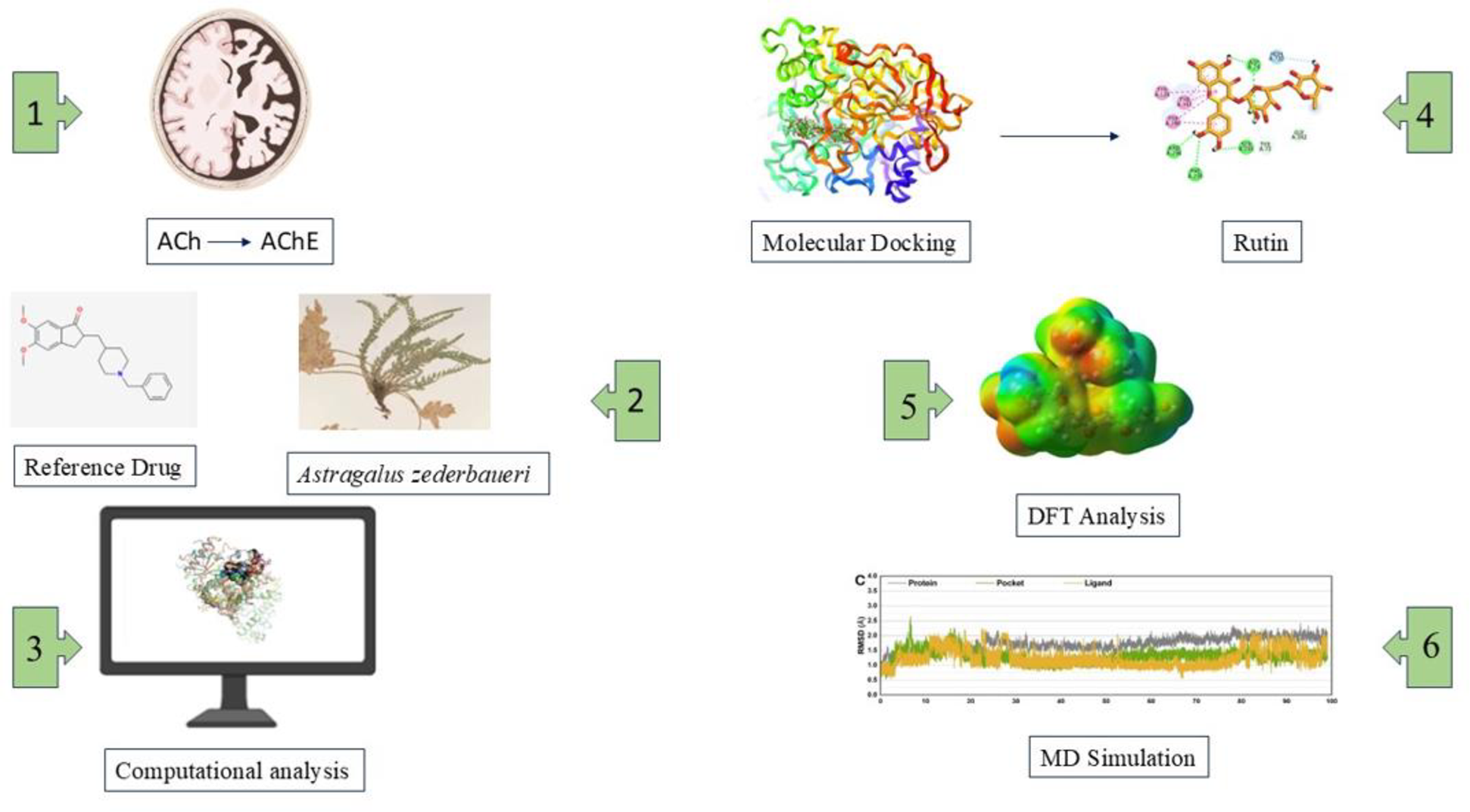

Supplement: S2 File — (TIF) [file pone.0346177.s006.tif]
