# Supplementary material for: Phytochemicals from Astragalus zederbaueri as Acetylcholinesterase Inhibitors for Alzheimer’s Therapy
Source: PLoS One. 2026 Apr 10;21(4):e0346177. doi: 10.1371/journal.pone.0346177 (PMC13068338; doi:10.1371/journal.pone.0346177)

**Supplementary Figure S2.** The heatmap of molecular properties highlights key attributes of the studied compounds


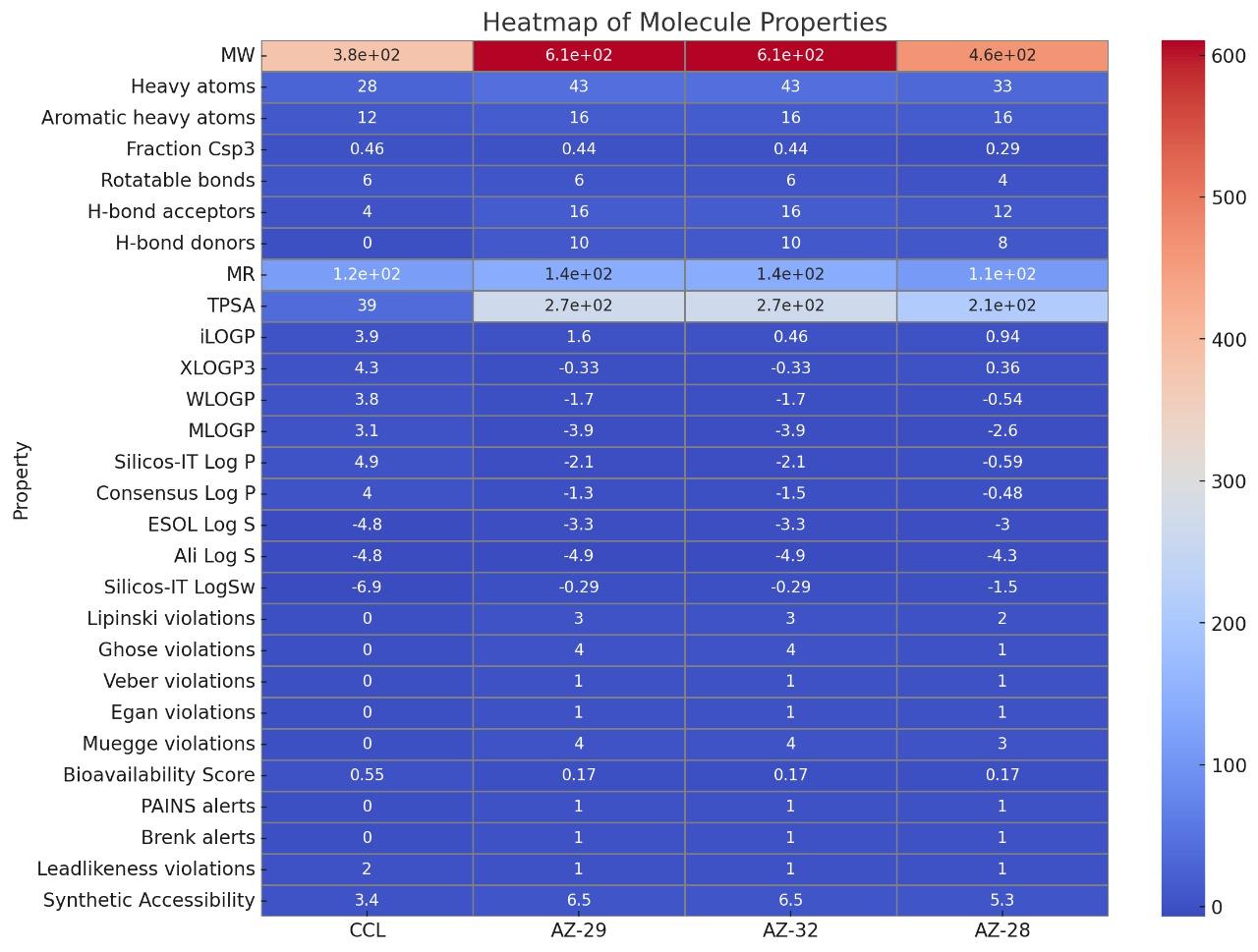

Supplement: S2 Fig — (DOCX) [file pone.0346177.s005.docx]
